# Manner/result polysemy as contextual allosemy: Evidence from Daakaka

**DOI:** 10.1007/s11049-024-09616-6

**Published:** 2024-10-18

**Authors:** Jens Hopperdietzel

**Affiliations:** 1 Department of Linguistics and English Language, The University of Manchester, Oxford Rd, Manchester, M13 9PL UK; 2 Department of German Language and Literature I, University of Cologne, Albertus-Magnus-Platz, 50923 Cologne, Germany

**Keywords:** Root meaning, Syntax/semantics interface, Manner/result polysemy, Contextual allosemy, Oceanic languages, Daakaka

## Abstract

Manner/result polysemy describes a phenomenon where a single root can encode both manner and result meaning components of an eventive verbal predicate. It therefore poses a challenge to (i) the hypothesis of manner/result complementarity as a fundamental constraint on verb/root meaning and (ii) a strict one-to-one mapping between roots and meaning. Examining novel data from the Oceanic language Daakaka, I provide further evidence that polysemous verbs like *tiwiye* ‘press manually, break’ only apparently violate manner/result complementarity, as manner and result meaning components are in complementary distribution. As both meaning components are sensitive to their morphosyntactic environment, I develop an account of contextual root allosemy, in which manner and result interpretations are associated with designated syntactic positions in relative configuration to an event-introducing verbalizer *v*. In particular, I argue that a single root may be associated with two non-compositional entries in the encyclopaedia, an eventive and a stative one, which allows the root to be merged in either the manner or result position. Independent support comes from suppletive verb forms in the paradigm of polysemous roots in Daakaka, where the spell-out conditions of contextual allomorphy and contextual allosemy overlap. Finally, I discuss theoretical and empirical challenges for alternative accounts of manner/result polysemy, including accounts based on derivation, coercion, and homophony.

## Introduction

In the study of root meaning, manner/result complementary represents a strong hypothesis of the lexicalization of meaning components in (verbal) predicates, according to which manner and result meaning components are in complementary distribution, i.e. roots of monomorphemic verbs encode either the manner or the result component of an event but not simultaneously (Rappaport Hovav and Levin 2010, 1998; Levin and Rappaport Hovav 1991). Due to their complementary distribution, roots are commonly grouped into two broad ontological classes that determine their semantic function, i.e. manner roots like $\sqrt{\mathit{hammer}}$ specify the manner component of manner verbs like *hammer*, whereas result roots like $\sqrt{\mathit{flat}} $ specify the result component of result verbs like *flatten*. While various (cross-linguistic) studies support the general tendency of the hypothesis (Levin 2015; Gast et al. 2014; Talmy 2000), potential counterexamples have evoked a controversial discussion about its universal status (Goldberg 2010; Beavers and Koontz-Garboden 2020, 2012; Ausensi et al. 2021; but see Husband 2011; Rappaport Hovav 2017) and its exact theoretical implementation (cf. Rappaport Hovav and Levin 2010; Mateu and Acedo-Matellan 2012; Alexiadou et al. 2015; Folli and Harley 2020).

In this paper, I approach this discussion from the perspective of the understudied language Daakaka (Northern/Central Vanuatu, Oceanic, Austronesian) where a group of transitive verbs is polysemous in lexicalizing either a manner or result meaning component: the verb *tiwiye*, for example, denotes ‘an attempt of an agent to break something by applying manual force on its end (∼ a proto-typical ‘breaking action,’ which will be glossed as ‘press manually’ for convenience)’ without the entailment that the object actually breaks.

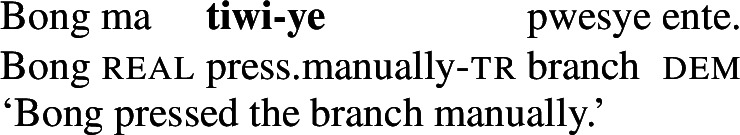

Yet, *tiwiye* can also denote ‘a broken (result) state’ without specifying the causing event, when it occurs in the non-initial position of resultative serial verb constructions (RSVCs, cf. Hopperdietzel 2020c).

(2)
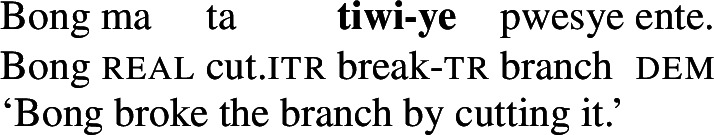

By the application of manner/result diagnostics (Rappaport Hovav and Levin 2010; Beavers and Koontz-Garboden 2020*inter alia*), I show that polysemous verbs in Daakaka do not entail manner and result meaning components simultaneously (cf. Levin and Rappaport Hovav 2014, 2013 on English *cut* and *clean*). As such verbs thus obey manner/result complementarity, they provide novel cross-linguistic evidence that manner/result polysemy does not necessarily falsify the hypothesis, as previously demonstrated by Levin and Rappaport Hovav (2013, 2014) for English, and Alexiadou and Anagnostopoulou (2013) for Greek.

However, the existence of manner/result polysemy raises non-trivial questions about the implementation of manner/result complementarity as a general principle of verb/root meaning, as it suggests that this principle operates neither on the root nor verb level. Consequently, it challenges traditional approaches that assume a strict binary classification of ontological verb/root classes (e.g. Rappaport Hovav and Levin 2010; Alexiadou et al. 2015), as polysemous roots seem to be underspecified, i.e. they are not restricted in their distribution.

(3)
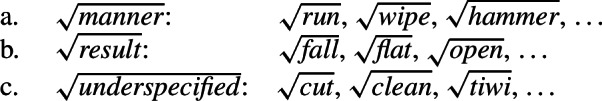

Examining the morphosyntactic properties of manner and result variants of polysemous verbs in Daakaka, I take manner/result polysemy to support an allosemy account of root meaning, in which the interpretation of underspecified roots is sensitive to the morphosyntactic environment in which the root appears (Levinson 2010; Mateu and Acedo-Matellan 2012; Alexiadou and Anagnostopoulou 2013; cf. Harley and Noyer 2000; Arad 2005; Borer 2005; Acquaviva 2009; Acedo-Matellan and Mateu 2014; Harley 2014; Pross 2019). More precisely, if a root is merged in the modifier position of an eventive verbalizer *v*, it receives a manner interpretation, whereas if a root is merged in the complement domain of *v*, it receives a result interpretation (cf. Alexiadou and Lohndal 2011; Folli and Harley 2020; Hopperdietzel 2022). Manner/result complementary then follows from the assumption that a single root cannot occupy two syntactic positions simultaneously and a single categorizer (here: the verbalizer *v*) can only categorize a single root at a time (Mateu and Acedo-Matellan 2012; cf. Embick 2010).

(4)

To account for the fact that many roots are restricted in their distribution, i.e. they occur in either the manner or result position, I argue that the morphosyntactic distribution of roots is ultimately restricted by the encyclopaedic entries they are associated with: eventive (manner) roots as event modifiers are uninterpretable in the result position; stative (result) roots as state modifiers are uninterpretable in the manner position (5a/5b). Polysemous roots, however, are associated with multiple allosemes, an eventive and a stative one, whose spell-out conditions are sensitive to their local morphosyntactic environment, i.e. they are subject to contextual root allosemy (5c) (Levinson 2010; Marantz 2013; Harley 2014). Roots more generally are thus uninterpretable in isolation and receive their idiosyncratic meaning only in the relative syntactic configuration to their categorizer.

(5)
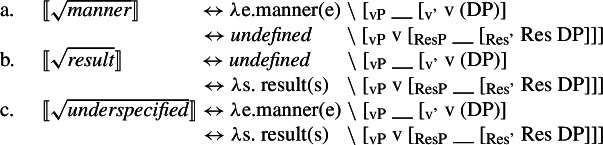

Crucial evidence for a morphosyntactic approach to root meaning comes from suppletive intransitive verb forms in the paradigm of Daakaka polysemous verbs that are sensitive to the presence of a Voice head: While the unergative manner variant of *tiwiye* ‘press manually, break’ is non-suppletive being realized as *tiwir* ‘press-manually (6a), the Voice-less unaccusative result variant *setyup* ‘break’ shows root suppletion (6b) (cf. von Prince 2015).

(6)
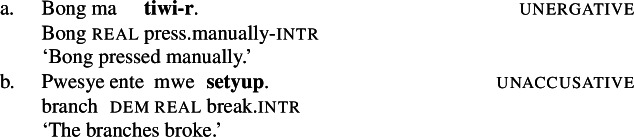

With VoiceP as the local domain for contextual allotypy (Marantz 2013; Harley 2014; Wood 2015; cf. Harðarson 2021), I demonstrate that while contextual allomorphy and contextual allosemy are predicted to overlap under the allosemy analysis, it challenges alternative analyses based on derivation, coercion, or homophony.

(7) $[\!\![\sqrt{\mathit{tiwi}}]\!\!]$ ↔ *λ*e. press.manually(e) | /tiwi/
  ∖ [_VoiceP_ DP [_Voice’_ Voice [_vP_ __ [_v’_ *v* DP]]]]
  ↔ *λ*e. press.manually(e) | /tiwi/
  ∖ [_VoiceP_ DP [_Voice’_ Voice [_vP_ __ [_v’_ *v* ]]]]
  ↔ *λ*s. broken(s) | /tiwi/
  ∖ [_VoiceP_ DP [_Voice’_ Voice [_vP_ *v*[_ResP ____ [_Res’_ Res DP]]]]]
  ↔ *λ*s. broken(s) | /setyup/
  ∖ [_vP_ *v* [_ResP ____ [_Res’_ Res DP]]]

This paper is structured as follows: Sect. 2 provides a background on the distribution of manner and result meaning components in verb meaning from an English perspective. Section 3 presents a detailed study of polysemous verbs in Daakaka, based on the application of more general and language specific manner/result diagnostics. This study reveals that both meaning components are in complementary distribution, providing novel cross-linguistic evidence that manner/result polysemy does not necessarily challenge the hypothesis of manner/result complementarity. Section 4 develops an allosemy analysis of manner/result polysemy in which manner and result interpretations are linked to a single root via contextual allosemy. This analysis is then independently supported by suppletive forms in the paradigm of polysemous verbs. Section 5 shows how alternative analyses cannot readily account for manner/result polysemy in Daakaka and beyond. Section 6 concludes.

## The distribution of manner and result meaning

In lexical semantics, verbal predicates are usually classified regarding their abstract event and argument structure, such as *activities*, *states*, or *accomplishments*, into which idiosyncratic roots are inserted (Vendler 1967; Dowty 1979; Rappaport Hovav and Levin 1998; Beavers and Koontz-Garboden 2020). Roots however have been argued to be unevenly distributed over such verb classes and to split into two broad ontological classes according to whether they encode manner or result meaning components of an abstract event (i.e. manner/result complementarity; Rappaport Hovav and Levin 2010*inter alia*; but see Beavers and Koontz-Garboden 2020).

In this section, I first introduce the (de-)compositional approach to event structure before illustrating manner/result complementarity in English with the help of morphosyntactic and semantic diagnostics that are sensitive to the presence of manner or result meaning components. I then turn to the phenomenon of manner/result polysemy, in which a single root may encode manner and result meaning, highlighting that such examples do not necessarily challenge the general intuition behind the hypothesis (Levin and Rappaport Hovav 2013, 2014), but raise more general questions about its nature.

### Event (de-)composition

In de-compositional approaches to event structure, verbal predicates are built from event structure templates based on grammatical primitives (structural verb meaning) into which roots are inserted (lexical or idiosyncratic verb meaning; cf. Dowty 1979; Rappaport Hovav and Levin 1998; Beavers and Koontz-Garboden 2020). There are two atomic eventualities that cannot be further decomposed; namely *activities* and *states*: on Rappaport Hovav and Levin’s (1998) account, an activity is formed by the modification an abstract ACT(ion) event with a (manner) root like $\sqrt{\mathit{hammer}}$ (8a), whereas a state is denoted by a (stative) root like $\sqrt{\mathit{flat}}$ (8b) itself.

(8)
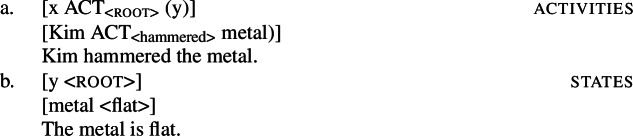

From these atomic eventualities and additional semantic operators, more complex eventualities can be derived (see Koontz-Garboden 2012 on the monotonicity of this process). Causative verbs like *flatten* are thereby decomposed into an (action) event and a (result) state that are in a causative relation which is introduced by a CAUSE operator (cf. Lewis 1973).^1^ Note that the result root solely denotes the result state leaving the nature of the causing event underspecified.

(9) [[x ACT] CAUSE [BECOME [y <root>]]] caus. accomplishments
  [[Kim ACT] CAUSE [BECOME [metal <flat>]]
  Kim flattened the metal.

To further specify the causing event by an additional manner root, resultative predicates are formed by a manner verb and a secondary adjectival result predicate entering a causative relation in a complex accomplishment predicate (cf. Beavers 2012 for an overview).

(10) [[x ACT_<root>_] CAUSE [BECOME [y <root>]]]
  res. accomplishments
  [[Kim ACT_<hammer>_] CAUSE [BECOME [metal <flat>]]]
  Kim hammered the metal flat.

According to this type of (de-)compositional approach, there are therefore two potential ways for roots to be integrated into the event structure of verbal predicates, namely as event modifiers of an abstract ACT(ion) event specifying the manner of the event, or as event arguments of the causative operator, naming the result state of an otherwise underspecified causing event.

### Manner/result complementarity

In the study of the distribution of root meaning, Rappaport Hovav and Levin (1998 et seq.) suggest that roots can *either* function as event modifiers *or* event arguments. A monomorphemic verb therefore cannot lexicalize both a manner and a result meaning component at the same time. Instead, the intended meaning must be expressed by bi-morphemic constructions, such as resultative accomplishments, as in illustrated in (10) above.

(11) * [[x ACT_<root1>_] CAUSE [BECOME [y <root_1_>]]]

This observation has been formulated as the hypothesis of manner/result complementarity which makes strong predictions about possible verb meanings in natural language.

(12) **Manner/result complementary**
  Manner and result meaning components are in complementary distribution: a verb lexicalizes only one. (Levin and Rappaport Hovav 2013, 50)

While subsequent studies show that this hypothesis reflects a general tendency in meaning distribution cross-linguistically (Alexiadou and Anagnostopoulou 2013; Gast et al. 2014; Levin 2015; Hopperdietzel 2020c; cf. Talmy 2000), there is an ongoing debate about its exact grammatical implementation and its universal status with potential counterexamples discussed in the literature (cf. Beavers and Koontz-Garboden 2012, 2017; Ausensi et al. 2021*inter alia*; see Rappaport Hovav 2017 for a response).^2^

As a consequence, individual verbs can be classified depending on the meaning component that is encoded by their underlying roots, i.e. the manner of an (action) event or the properties of a (result) state. As most roots can only encode either a manner or result meaning component, roots are split into (at least) two broad ontological classes, manner and result roots, which determine their distribution as event modifiers and event arguments in the decomposed verbal structure (for the distinction between stative and result roots see fn. 6; Rappaport Hovav and Levin 2010; Alexiadou et al. 2015; Rappaport Hovav 2017).

(13)
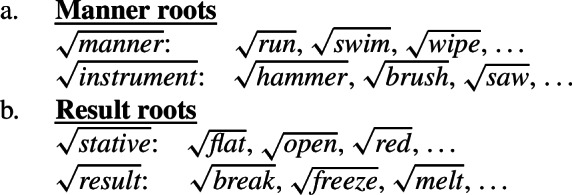

The semantic difference between manner and result has thereby been linked to the scalar properties of the verbal predicates in question (Rappaport Hovav and Levin 2010; Beavers and Koontz-Garboden 2012; Rappaport Hovav 2014; but see Beavers and Koontz-Garboden 2017 for a critical discussion): result verbs denote *scalar changes*, i.e. “a change in the value of a scalar attribute [of an argument] in a particular direction, where the direction is determined by the polarity of the scalar base” (Rappaport Hovav 2014, 264). Manner verbs instead denote *non-scalar changes*, i.e. “any change that cannot be characterized in terms of an ordered set of degrees along a dimension representing a single attribute (Rappaport Hovav 2014, 273). Manner/result complementarity can therefore be determined by diagnostics that are sensitive to the presence of a manner or result meaning component and their associated (non)scalar change (Rappaport Hovav and Levin 2010; Beavers and Koontz-Garboden 2012*inter alia*).^3^

On the one hand, manner verbs like *hammer* (14a) but not result verbs like as *flatten* (14b) can appear in conative constructions where the object is realized within a locative PP. As conative constructions necessarily presuppose non-scalar changes (cf. Guerssel et al. 1985; Levin and Rappaport Hovav 1991; Goldberg 1995), result verbs which lexicalize scalar changes require the undergoer of the scalar change, i.e. the holder of the resulting state, to be realized as a direct argument of the predicate (Rappaport Hovav 2014; also Levin 2017).

(14)
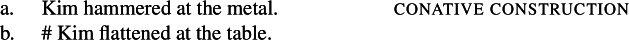

On the other hand, result verbs can appear in anticausative constructions (15b), in which an external argument is syntactically and semantically absent (Levin and Rappaport Hovav 1995; Alexiadou et al. 2006). As verbs that lexicalize a manner component often require an agent argument that performs the action denoted by the root, only verbs that lexicalize a result component are felicitous in this context (15a) (Alexiadou 2015; also Levin 2017).^4^

(15)
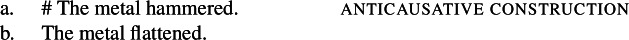

Further evidence comes from the felicity of a denial of a change in the context of manner but not of result verbs (Rappaport Hovav and Levin 1998; Beavers 2011; Beavers and Koontz-Garboden 2012). If a verb lexicalizes a result component, a denial of change into the particular result state leads to a contradiction, due to the scalar change entailed by the verb (16b). In contrast, if a verb lexicalizes a manner component, a denial of result is felicitous (16a).^5^

(16)
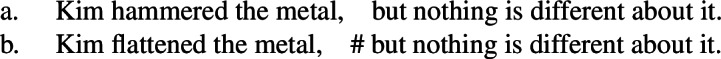

The complementary distribution of manner and result roots therefore suggests that type-theoretically, roots differ in their semantic type: While manner roots denote specific dynamic eventualities of type *e*, result roots denote stative eventualities of type *s* (Harley 2005; Embick 2009; cf. Levinson 2014).^6^

(17)
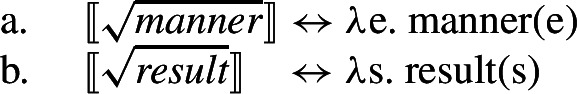

As a result, the complementary distribution of manner and result can be interpreted to follow from the semantic type of the respective root which restricts their function as either event modifiers, i.e. eventive manner roots, or event argument, i.e. stative result roots, in the decomposed structure of verbal predicates (cf. Ausensi 2021 for a similar intuition).

### Manner/result polysemy

A challenge for the hypothesis of manner/result complementarity comes from verbs that are sensitive to both manner and result diagnostics, and therefore appear to lexicalize both a manner and a result meaning component simultaneously (cf. Guerssel et al. 1985; Levin 1993 on *cut*-type verbs). In English, the verb *cut*, for example, patterns with both manner and result verbs in being felicitous in both the conative (18a) and the anticausative construction (18b) (Levin and Rappaport Hovav 2013).

(18)
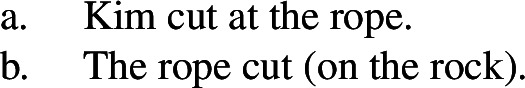

However, a careful examination of the properties of *cut* reveals that the verb may lexicalize a manner or a result component, but crucially not at the same time, as the manner component drops out in its result variant and vice versa (Levin and Rappaport Hovav 2013). Therefore, a denial of a result state is only felicitous when *cut* occurs in the conative construction, but not when it occurs in the anticausative construction (cf. Beavers 2010)

(19)
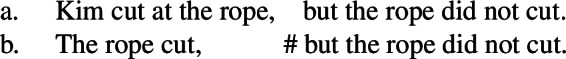

Based on the distribution of manner entailments in the respective contexts, Levin and Rappaport Hovav (2013) argue that *cut* is polysemous, and has two distinct meanings associated with its manner and result variant. In the conative construction, *cut* necessarily entails ‘an action performed by an agent with a sharp-bladed instrument,’ as indicated by its infelicity in contexts where no such action is involved (20a).

(20)
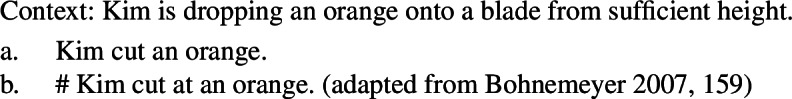

In its result use, neither an action nor a sharp-bladed instrument is required for the felicity of *cut* (20b), which instead has the interpretation of ‘a clean separation,’ as illustrated by the anticausative construction in (21).

(21) The rope cut on the rock.

As a consequence, polysemous verbs like *cut* do not (necessarily) falsify the hypothesis of manner/result complementarity, as they denote either the manner or the result of an event, but not at the same time (see also Glass 2021 on *lift*, Levin and Rappaport Hovav 2014, 2013 on *clean* and *climb*, but see Beavers and Koontz-Garboden 2017 for a critical discussion).

However, manner/result polysemy raises non-trivial questions about the level on which manner/result complementarity as a lexicalization principle operates: on the one hand, polysemous verbs seem to falsify the rigid binary classification of root classes into manner and result roots, as they suggest that roots can be underspecified with respect to their ontological class.

(22)
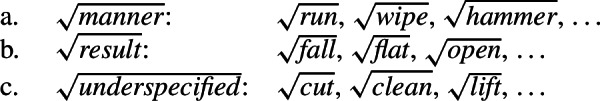

On the other hand, polysemous verbs challenge a one-to-one mapping between root and encyclopaedic meaning. As manner and result meaning of the same root do not entail each other and refer to distinct types of eventualities (*event* vs. *state*), they must be independently stored in the encyclopaedia. A postulation of two independent homophonous roots as in (23) however risks blurring the intuitive relation of the two variants of polysemous verbs.

(23)
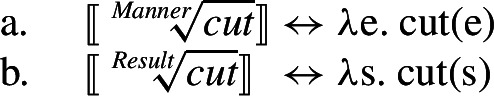

Based on the examination of novel morphological evidence from polysemous verbs in the Oceanic language Daakaka, I will show that manner/result complementarity ultimately operates on the syntactic level (cf. Mateu and Acedo-Matellan 2012), which suggests a one-to-many mapping between roots and their encyclopaedic meaning.

## Manner/result polysemy in Daakaka

While previous studies focused on data from better-studied Indo-European languages, including English, German (Gamerschlag et al. 2014), Greek (Alexiadou and Anagnostopoulou 2013), and Italian (Folli and Harley 2020), as well as Hebrew (cf. Segal and Landau 2012), I present novel data from the understudied Oceanic language Daakaka which indicates that manner/result polsemy is a widespread cross-linguistic phenomenon. In the following, I focus on the polysemous verb *tiwiye* ‘press manually, break’ (24).^7^

(24)
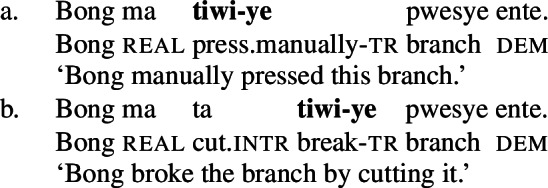

By the application of morphosyntactic and semantic diagnostics, I demonstrate that these verbs lexicalize a manner component when they occur as independent verbs as well as in the initial (manner-denoting) position of resultative serial verb constructions (RSVCs), but a result component when they occur in the non-initial (result-denoting) position of RSVCs. Crucially, the distributional restriction of the result variant to the non-initial position in RSVCs reflects a more general property of causative verbs in Daakaka (Hopperdietzel 2021b, 2020c).

I begin this investigation with a brief background on the language and the methodology used in data collection, before applying morphosyntactic and semantic diagnostics that are sensitive to the lexicalization of manner and result meaning components. This investigation reveals that such meaning components are in complementary distribution and correlate with the syntactic position of the verb. Finally, I demonstrate that such distributional restrictions are shared with other (non-polysemous) manner and (causative) result verbs in Daakaka.

### Typological and methodological background

Daakaka (Northern/Central Vanuatu, Oceanic, Austronesian) is spoken by a relatively small community (∼1,000 speakers) on the island of Ambrym, which is part of the Vanuatu archipelago in the Western part of the Pacific Ocean (von Prince 2015). Due to its small speaker population and the growing influence of Vanuatu’s *lingua franca* Bislama, the language qualifies as endangered. Daakaka is closely related to its neighbouring languages on the island Daakie (Krifka 2011) and Dalkalaen (von Prince 2012), as well as North Ambrym (Franjieh 2012).

The basic word order is SVO. Verbal predicates show preverbal subject agreement and mood-prominent TMA marking as well as verb-final transitivity marking (von Prince 2015; Hopperdietzel 2020b, 2021b). Noun phrases are not overtly marked for case, optionally marked for number, and may be dropped in discourse.

(25)
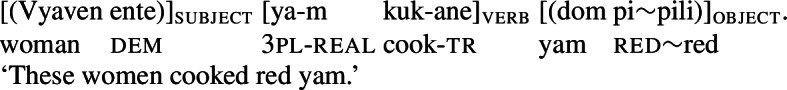

The majority of the data presented in this paper comes from elicitation sessions (2017–2022) in Emyotungan and Port Vila with three native speakers (all male; Age: 23, 30, and 61) who were born and raised in the village of Emyotungan on Ambrym. The data was collected via storyboard and picture-based elicitation as well as judgement tasks based on a self-designed questionnaire that targets manner and result meaning. The material and data are partly accessible at the Kaipuleohone Language Archive at the University of Hawai’i at Manoa (Hopperdietzel 2020a). Additional data was extracted from available sources, which include a comprehensive grammar (von Prince 2015), a dictionary (von Prince 2017) and a corpus of oral speech data (68,291 tokens; von Prince 2013).

### Manner/result diagnostics

As illustrated in Sect. 2.2, the distribution of manner and result meaning components in the event structure of verbal predicates is sensitive to certain morphosyntactic and semantic diagnostics. In this section, I apply such diagnostics to verbal predicates in Daakaka, focusing on the polysemous verb *tiwiye* ‘press manually, break.’ This includes (i) instrumental modification, (ii) object theta roles, (iii) object deletion, (iv) denial of result, and (v) the distribution of the verbs in Daakaka RSVCs.^8^ The investigation reveals that the distribution of manner and result meaning components correlates with the syntactic position of the verbal predicate: if *tiwiye* functions as an independent predicate or occurs in the initial position of RSVCs, it lexicalizes a manner component; if it occurs in the non-initial position of RSVCs instead, it lexicalizes a result component. Consequently, manner and result meaning components are in complementary distribution, in line with manner/result complementarity.

#### Instrumental modification

A first diagnostic comes from the distribution of instrumental modifiers which is sensitive to the presence of a manner component (cf. Talmy 1976; Croft 1991; Dowty 1991; van Valin and Wilkins 1996; Beavers and Koontz-Garboden 2012; Rissman et al. 2015): in the context of manner verbs, instrumental modifiers must satisfy the manner component lexicalized by the verbal predicate, i.e. the instrument must be compatible with the action denoted by the root (Kiparsky 1997; Harley 2005; Bleotu and Bloem 2020). This contrasts with result verbs where instrumental modifiers are not constraint by the causing action, as long as they are compatible with the result state denoted by the root.^9^ In English for example, the manner component of the manner verb *hammer* can be satisfied by an instruments like *a shoe* or *a hammer*, but (proto)typically not by a *steam roller*, whereas the result verb *flatten* can combine with all three kinds of instruments.

(26)
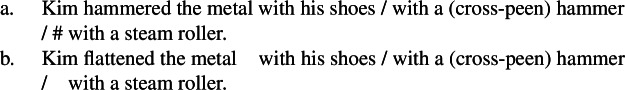

In Daakaka, instrumental modification of the polysemous verb *tiwiye* is sensitive to the syntactic context in which the verb appears: as an independent predicate, *tiwiye* necessarily refers to a *manual* action, which is why instrumental modifiers like *ane tee* ‘with an axe’ are infelicitous.

(27)
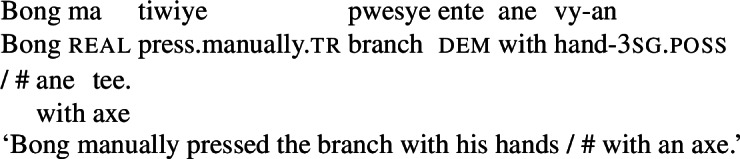

If *tiwiye* however occurs in the non-initial position of RSVCs, instrumental modifiers like *ane tee* become felicitous, suggesting that the manner component that is lexicalized by *tiwiye* in its independent use drops out. Instead, instrumental modification is now sensitive to the manner component of initial manner verb, as indicated by the infelicity of the instrumental modifier *ane vyan* ‘with his hand’ in the context of the manner verb *ta* ‘cut.’ Here, Daakaka *ta* differs from English *cut* in that it does entail, and not only imply, the use of a sharp instrument and exhibits the properties of other manner verbs (see also Sects. 3.2.3 and 3.2.4).

(28)
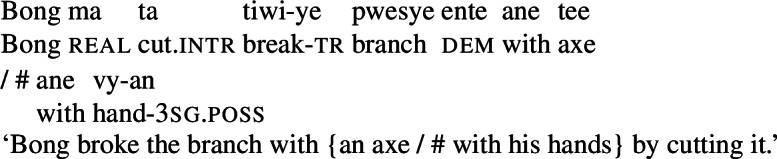

The distribution of instrumental modifiers therefore suggests that *tiwiye* only entails a manner component when it occurs as an independent predicate.

**Fig. 1:**
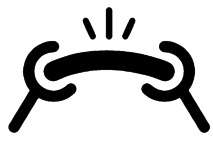
Stereotypical illustration of the manner variant of *tiwiye* ‘manual pressing.’^10^

**Fig. 2:**
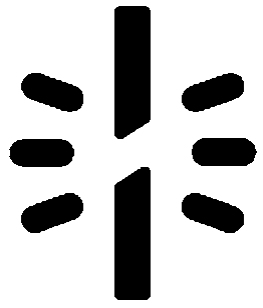
Stereotypical illustration of the result variant of *tiwiye* ‘break.’^11^

The difference between the two readings of *tiwiye* can be illustrated by the pictograms below: Manner *tiwiye* describes a manual action of an agent pressing on the both ends of typically long and thin object that the agent holds in this hands, e.g. a branch of a tree, a pen, or a shellfish, without entailing any effect on the object (Fig. 1). I will paraphrase this interpretation here roughly as ‘manual pressing.’ Result *tiwiye* instead describes a ‘breaking’ event without entailing the manner of the causing action (Fig. 2).

#### Object theta roles

The second diagnostic comes from theta roles that are assigned to objects, as direct objects of manner and result verbs differ in the theta role they receive (cf. Levin and Rappaport Hovav 1995; Kratzer 2005; Levin 2020): objects of manner verbs receive the patient role of the action event, whereas object of result verbs receive the holder role of the result state. Manner and result verbs can therefore vary in their compatibility with certain objects. In English for example, the object *the cup* cannot satisfy the patient role of the manner verb *pour*, which is restricted to liquids like *the tea*, but it can satisfy the holder role of the result verb *fill*.

(29)
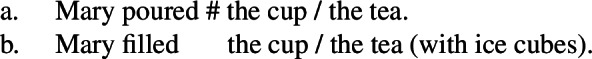

In Daakaka, the polysemous verb *tiwiye* necessarily selects for long thin objects, as the manner component restricts the patient role to objects that an agent can hold in his hands to perform a ‘manual pressing’ action. This is why an object like *pwesye ente* ‘the branch’ is felicitous while an object like *lee ente* ‘the tree’ is not (outside of fictional contexts involving giants or toy trees).

(30)
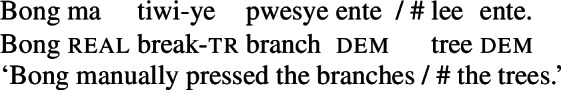

When *tiwiye* occurs as the non-initial predicate in RSVCs however, objects are not subject to the same restrictions, as long as the object can be construed as the holder of ‘broken’ state and satisfies the patient role of the initial manner verb. In contrast to its infelicity in (30), the object *lee ente* ‘the tree’ is now felicitous in the context of the manner verb *ta* ‘cut’ (31).

(31)
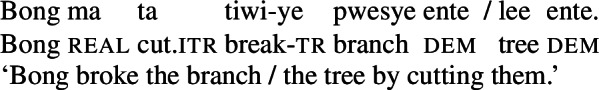

Consequently, *tiwiye* as an independent predicate differs from *tiwiye* in the non-initial position of RSVCs in its selectional restrictions on the direct object in the way that is expected for the contrast of manner and result verbs.

#### Object omission

The third diagnostic comes from the availability of object omission (Levin and Rappaport Hovav 2001; Wittek 2011; Alexiadou et al. 2014; but see Goldberg 2001; Mittwoch 2005). While scalar result verbs like *flatten* require direct object DP as the argument of the scalar change to be syntactically realized, non-scalar manner verbs like *hammer* are less restrictive and allow their object to be realized by a PP (as in the conative construction; see Sect. 2.2) or to be completely absent (33) (Rappaport Hovav and Levin 2010; Beavers and Koontz-Garboden 2012; Rappaport Hovav 2014; Levin 2017; cf. Beavers and Koontz-Garboden 2017 for further discussion).^12^

(32)
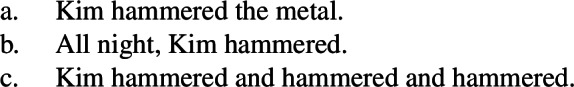

(33)
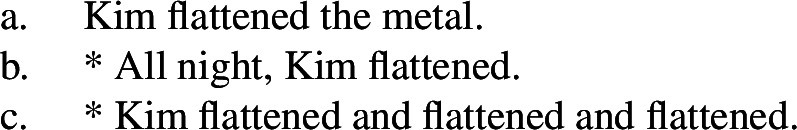

While the application of this diagnostic is quite transparent in languages such as English that allow pro-drop only in restricted contexts, Daakaka as a pro-drop language generally permits the omission of object arguments. However, Daakaka marks the syntactic representation of a (potentially covert) object by transitive morphology on the verb (von Prince 2015; Hopperdietzel 2020b,c). This is illustrated by the manner verbs *kolir* ‘sing’ and *ta* ‘cut’ below: In the presence of an overt object, the verb is obligatorily marked by transitive morphology, the productive transitive suffix *-ane* (34) or the suppletive form *te* (35) (see Sect. 5.3 on transitive morphology being sensitive to root class; cf. von Prince 2015; Hopperdietzel 2020b). In discourse prominent contexts however, the object may be dropped with a referential, specific interpretation. Yet, the underlying presence of a silent *pro* argument is still recoverable by transitivity marking on the verb (34b)/(35b).

(34)

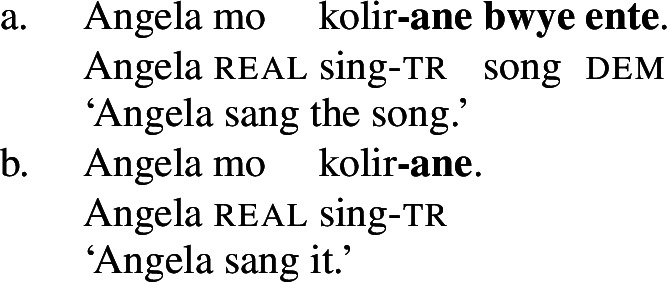

(35)
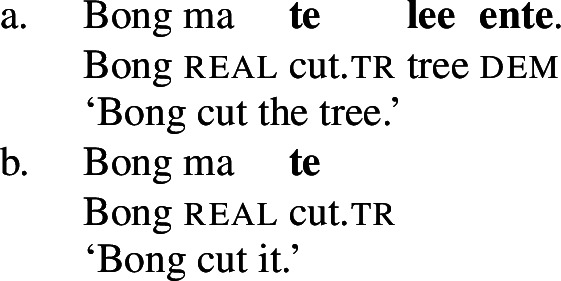

In contrast, the morphosyntactic absence of an argument is indicated by the absence of transitivity marking on the respective verbs which show up in their intransitive/unergative forms. Unlike *pro-*drop in the examples above, the omitted object receives a non-referential, unspecific interpretation (36)/(37).

(36)
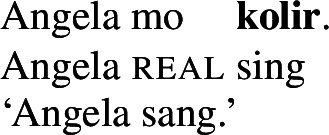

(37)
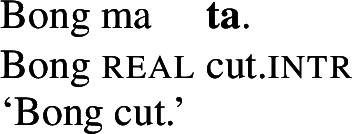

As predicted, only manner verbs like *kolir* ‘sing’ or *ta* ‘cut,’ but not result verbs can appear in such contexts, as the latter lack intransitive/unergative verb forms. The intransitive form of stative result verbs, *mwelili* ‘be.small’ and suppletive *lyoo* ‘be.split’ thus have stative/anticausative interpretations instead. Therefore, causative result verbs only allow object omission if the object is established in the previous discourse contexts, i.e. they must realize their direct object overtly or covertly (by *pro*), as indicated by obligatory transitive marking on the verb.

(38)

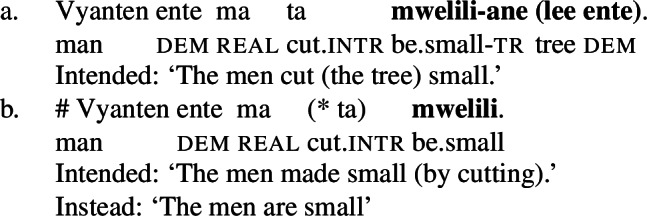

(39)
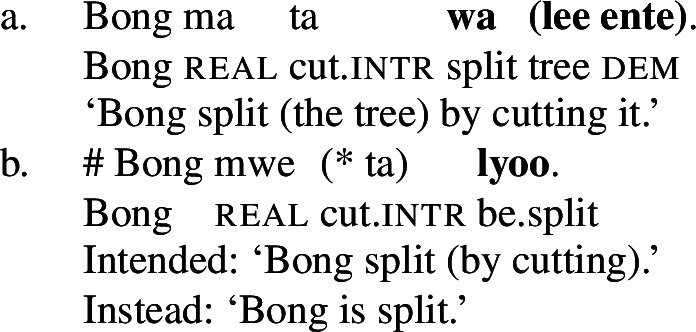

Unlike regular causative verbs like *mweliliane* ‘make small’ or w*a* ‘split,’ polysemous verbs exhibit an intransitive/unergative verb form which indicates that the omission of a (c)overt object argument is grammatical. In the intransitive/unergative form of *tiwiye* ‘press manually, break,’ the transitive marker *-ye* (40a/40b) is replaced by the intransitive marker *-r* (40).

(40)
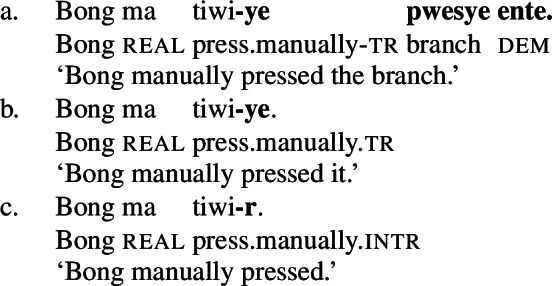

Crucially, however, the intransitive verb form *tiwir* is no longer polysemous and solely expresses the manner interpretation of *tiwiye*, i.e. a ‘manual pressing,’ as indicated by the absence of a stative/anticausative interpretation (which is realized by a suppletive verb form instead; see Sect. 4.4).

(41)
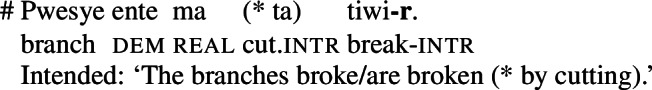

The distribution of intransitive/unergative verb forms of polysemous verbs in Daakaka therefore indicates that *tiwiye* has the properties of bi-eventive result verbs when it appears in the non-initial position of RSVCs.

#### Denial of result

Another diagnostic comes from the denial of result state which is felicitous only with mono-eventive manner verbs which do not entail a result state but not with bi-eventive causative verbs (see Sect. 2.2). This observation also holds for Daakaka: In a context where the action of the agent does not have any effect on the object, causative result verbs like *wa* but not manner verbs like *te* ‘cut’ are infelicitous.

(42)
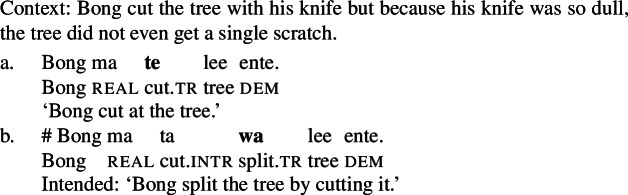

When polysemous verbs like *tiwiye* occur as independent predicates, they are felicitous in contexts where the object is not affected by the action performed by the agent (43a); when they occur in the non-initial position of RSVCs however, a denial of the result is contradictory and thus infelicitous (43b), which indicates that *tiwiye* lexicalizes a result component only in this construction.^13^

(43)
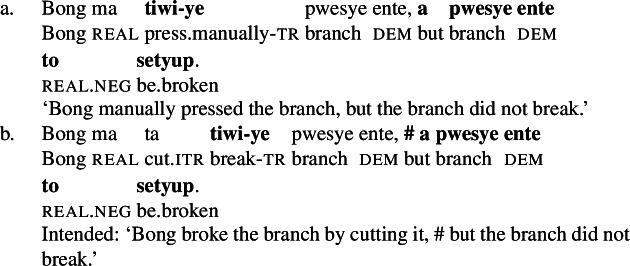

Therefore, this diagnostic further suggests that *tiwiye* is polysemous between a manner and result verb, which however is determined by its morphsyntactic context.

#### Resultative constructions

A final diagnostic comes from resultative constructions, in which both the means/manner and the result state of a (dynamic) event are encoded by distinct predicates. In English, resultative meaning can be expressed by either resultative secondary predication or means constructions (cf. Talmy 2000): In resultative secondary predication, a manner verb denotes the manner of an (action) event while the result component is realized by a non-verbal secondary result predicate (44a) (Halliday 1967; Simpson 1983; Beavers 2012; *inter alia*). In contrast, a causative result verb denotes the result state of an underspecified (action) event, which is further specified by a non-verbal manner adjunct in the means construction (44b) (Fodor 1970; Sæbø 2008; Hopperdietzel 2022*inter alia*).

(44)
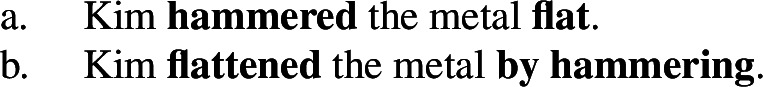

The distribution of secondary predicates is thereby sensitive to the lexicalization of a manner or result meaning component by the verbal main predicate. In resultative secondary predication, the verbal predicate denotes the manner of the action and can therefore combine with various result states denoting secondary predicates (Levin 2017; but see Goldberg 1995; Wechsler 2005 on scalarity and pragmatic compatibility).

(45)
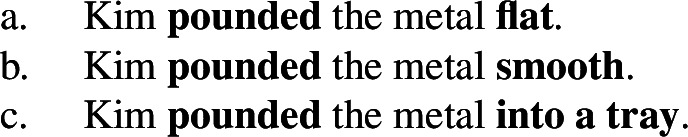

An additional manner modification by a means adjunct must satisfy the meaning component already lexicalized by the verbal main predicate, and is therefore much more restricted (cf. Williams 2015; Biggs and Embick 2020). In (46), a *pounding* action can be further specified as a *hammering* but not as a *driving* action.

(46)
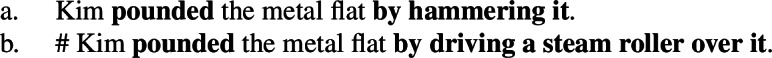

This contrasts with means constructions in which the verbal main predicate lexicalizes a result state which is caused by an underspecified (action) event. Due to its underspecificity, the entailed causing event can be modified by various manner-denoting secondary predicates.

(47)
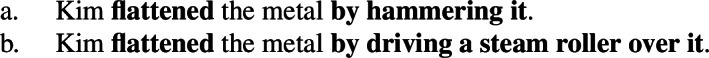

As the causative main predicate already encodes a result component, result verbs cannot enter true resultative secondary predication, as two distinct result states are infelicitous in a mono-clausal environment (Unique Path Constraint; Goldberg 1991; Tenny 1994; Levin and Rappaport Hovav 1995; but see Yu et al. 2023 for potential counterexamples). Instead, stative secondary predicates may only function as modifiers of the result already denoted by the verb in so-called *weak* resultatives (Washio 1997; cf. Kratzer 2005; Levinson 2010).

(48)
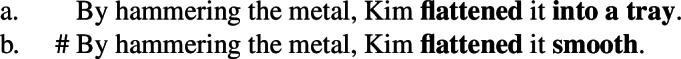

Therefore, the lexicalization of a manner or result component has an influence on the distribution of manner and result modification in resultative constructions (Beavers and Koontz-Garboden 2012).

In Daakaka, resultative meaning is primarily expressed by RSVCs, in which an intransitive manner verb in the initial position combines with a transitive causative result verb in non-initial position (49a) (Hopperdietzel 2021b, 2020c). Syntactically, the manner verb functions as a means adjunct to the causative result verb, which is the main predicate of the construction (see also Sect. 4.2). Crucially, the order of the manner- and result-denoting predicates is fixed, and indicates their ontological class (49b/49c).

(49)
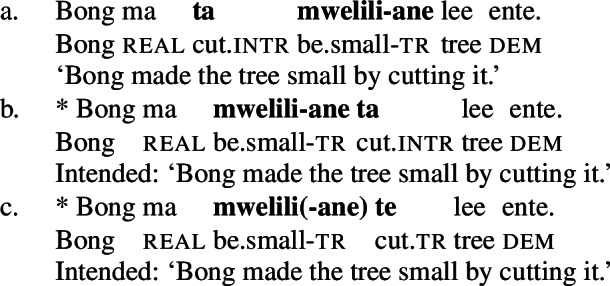

Like in English resultative constructions, result-denoting non-initial predicates can combine with various manner verbs in the initial position (50), and vice versa (51).^14^

(50)

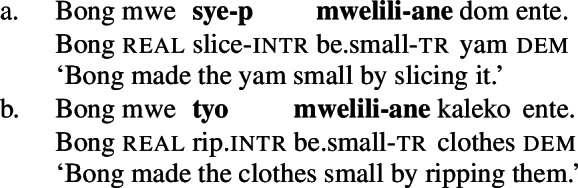

(51)
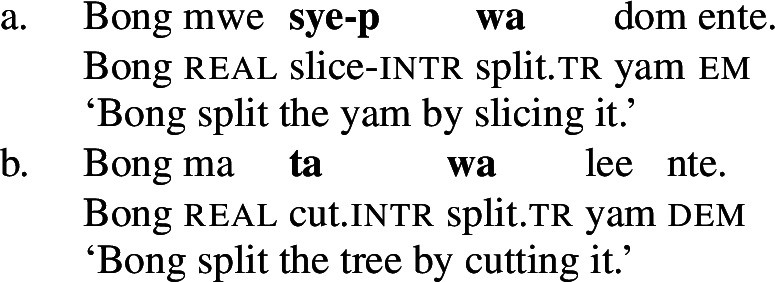

However, a further specification of the manner and result components is subject to compatibility restrictions, in that the modifiers must satisfy the meaning components already entailed by the main predicates, as illustrated for additional specification of the manner of the causing action in (52) and (adverbial) result modification in (53).^15^

(52)

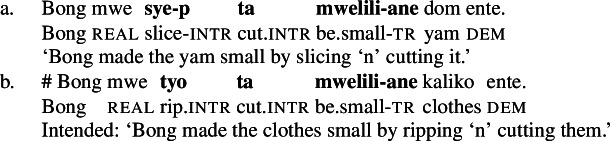

(53)
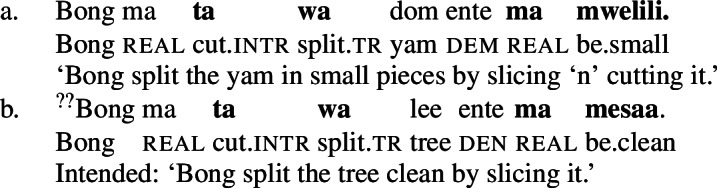

The distribution of verbal predicates and manner and stative modifiers therefore suggests that the initial position RSVCs is restricted to manner verbs whereas the non-initial position is restricted to result verbs.

Unlike pure manner and result verbs, such as *te* ‘cut’ and *mweliliane* ‘to make small’ respectively, polysemous verbs like *tiwiye* ‘press manually’ can appear in both positions. When *tiwiye* occurs in its intransitive form *tiwir* in the initial position of RSVCs, it denotes the manner of the causing event, i.e. a ‘manual pressing action,’ as indicated by the infelicity of non-manual instrumental modifiers like *ane tee* ‘with an axe’ (54a) and incompatible manner verbs like *ta* ‘cut’ (54b).

(54)
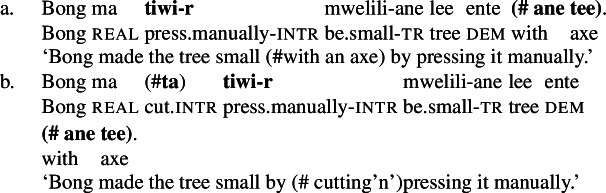

The felicity of various result-denoting non-initial verbs shows that *tiwir* does not license a result state, as expected for the intransitive/unergative verb form (see Sect. 3.2.3). Note that *tiwir* in combination with *veni* ‘kill’ can be used to describe the killing of small animals with a hard shell, e.g. insects or shellfish.

(55)
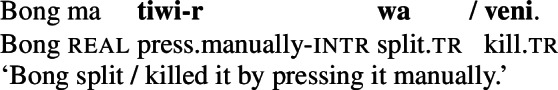

When *tiwiye*, in its transitive form, appears in the non-initial position of RSVCs, it solely denotes a ‘broken’ result state of the (action) event modified by the initial manner verb. Therefore, *tiwiye* is now compatible with instrumental modifiers like *ane tee* ‘with an axe’ (56a) and various manner verbs in RSVC-initial position (56b). Also, a further modification of the result state is restricted, as long as the result states are compatible (56c).

(56)
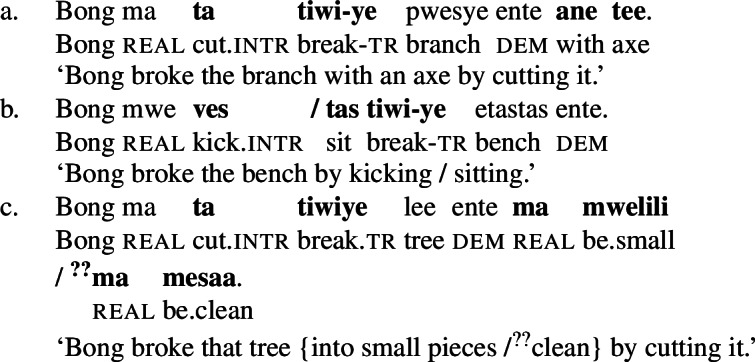

Crucially, both (intransitive) manner and causative result variants of polysemous verbs can co-occur in their respective positions in RSVCs without redundancy in meaning. Here, the initial *tiwir* solely denotes the manner of the causing action, i.e. a ‘manual pressing,’ while the non-initial *tiwiye* denotes its result, i.e. a ‘broken’ state.

(57)
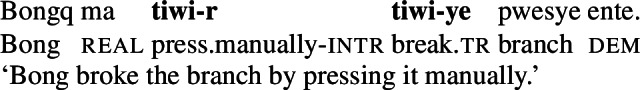

The distribution of *tiwiye* provides additional evidence for its polysemous status, as it cannot only occur in both the manner and the result denoting position but can also combine with its own counterpart.

#### Summary

The results of manner/result diagnostics in Table 1 indicate that *tiwiye* is subject to manner/result polysemy, as it shows properties of both manner verbs like *ta* ‘cut’ and result verbs like *wa* ‘split.’ As manner and result meaning components are in complementary distribution, Daakaka *tiwiye* obeys the hypothesis of manner/result complementarity. In addition, the Daakaka data suggests that manner/result polysemy is a widespread cross-linguistic phenomenon that can be found in a range of unrelated languages.

**Table 1:** Sensitivity of the polysemous verb *tiwiye* to manner/result diagnostics

	**Manner verbs** e.g. *ta* ‘cut’	**Manner** ***tiwiye*** ‘press manually’	**Result** ***tiwiye*** ‘break’	**Result verbs** e.g. *wa* ‘split
Restrictions on instrumentals	✓	✓	✗	✗
Restrictions on objects	(✓)	✓	✗	(✗)
Object deletion	✓	✓	✗	✗
Denial of result	✓	✓	✗	✗
Independent predicate	✓	✓	✗	✗
Initial position in RSVCs	✓	✓	✗	✗
Non-initial position RSVCs	✗	✗	✓	✓

However, the investigation also revealed that the interpretation of *tiwiye* as a manner or result verb is determined by its morphosyntactic context: While *tiwiye* denotes the manner of an action as an independent predicate and in the initial position of RSVCs, its result interpretation is restricted to the non-initial position of RSVCs. In the following, I demonstrate that this restriction follows from an independent language-specific constraint on causative verbs in Daakaka that are generally subject to a serializing condition.

### Serializing causatives

In Daakaka, causative result verbs differ from manner verbs in that they cannot function as independent predicates of a clause but need to combine with a manner predicate in RSVCs (Hopperdietzel 2021b, 2020c).

(58) **Serializing condition on causatives in Daakaka**
  If a verb denotes a causative relation between an event and a state, it must combine with a manner verb that specifies the causing event.
  (Hopperdietzel 2021b, 418)

This constraint holds for all types of causative verbs, including morphological causatives like *mweliliane* that are derived from stative property concept verbs like *mwelili* ‘to be small’ by the attachment of transitive morphology (59), which on its own does not contribute causative semantics, and lexical causatives like *wa* ‘split’ (60).^16^

(59)

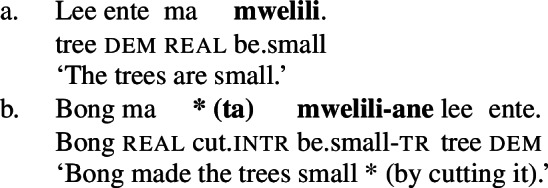

(60)
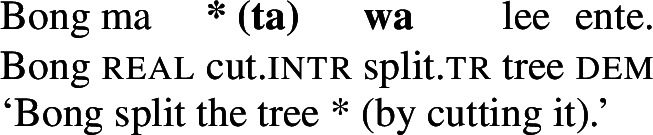

Even though lexical causative verbs are restricted to the non-initial position of RSVCs, they show verb-specific properties that distinguish them from non-verbal predicates: On the one hand, causative verbs like *wa* reduplicate to indicate a pluractional semantics, a process that is restricted to verbal predicates in Daakaka (Hopperdietzel 2020c; cf. von Prince 2015).

(61)
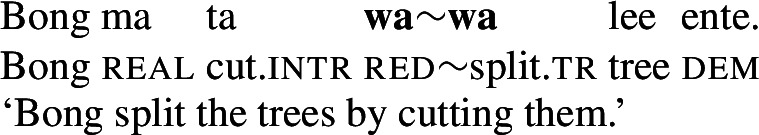

On the other hand, they exhibit (often suppletive) intransitive stative/anticausative verb forms which denote (the change into) the state entailed by the lexical causative verb.^17^

(62)
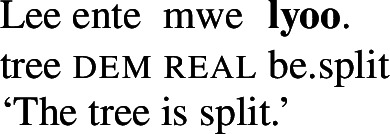

Finally, they contrast with non-verbal predicates like property concept adjectives such as *towo* ‘big,’ in that only the latter can be used together with the predicative copula *i* (von Prince 2015).

(63)
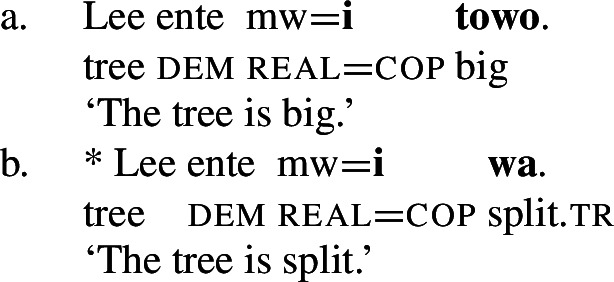

Despite the bound nature of causative verbs, Hopperdietzel (2021b, 2020c) argues for their main predicate status in RSVCs. Morphosyntactic evidence for this assumption comes from the distribution of transitive morphology, as illustrated in (64) where the initial manner verb is marked by the intransitive suffix *-p*, while the non-initial causative result verb is marked by the transitive suffix -*ane* (see Sect. 3.2.2).

(64)
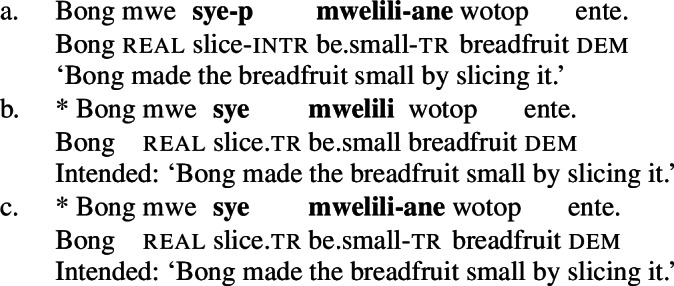

The presence of transitive morphology on the non-initial predicate suggests that both arguments are in a local configuration with the causative result verb (Hopperdietzel 2020b; cf. Deal 2010; Nie 2017), whereas the initial manner verb only introduces an agentive argument. Daakaka RSVCs therefore mirror the morphological pattern of English means constructions in which the causative result verb functions as the transitive main predicate of the clause (see Sect. 3.2.5; cf. Hopperdietzel 2022 for a typological overview).^18^

Semantic evidence supports the morphosyntactic analysis: notably, Daakaka RSVCs resemble English means constructions regarding the availability of a narrow repetitive reading of repetitive modifiers like *again* where the repetitive modifier solely scope over the causing event to the exclusion of the result state. As illustrated in (65), such a reading is only available in means constructions when the repetitive modifier attaches within the means adjunct but not in resultative secondary predication where the manner verb functions as the main predicate (von Stechow 1996; Lechner et al. 2015; Hopperdietzel 2022).

(65)
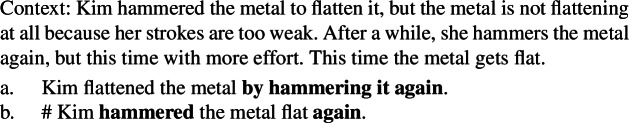

Crucially, Daakaka RSVCs are felicitous in narrow repetitive contexts, indicating that they have the underlying properties of means constructions, in that the manner verb adjoins to the causative result verb.

(66)
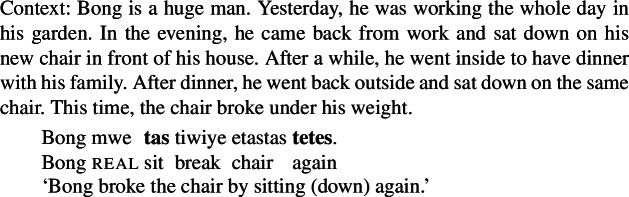

While the unexpected obligatory nature of the adjoined manner verb requires further investigation, the crucial point here is that causative result verbs are subject to a serializing condition in Daakaka and must co-occur with an adjoined manner verb.^19^ The serializing condition of the result interpretation of polysemous *tiwiye* ‘press manually, break’ therefore follows from more general language specific constraints on event structure. Consequently, the result variant of *tiwiye* has properties of other causative verbs in the language, and it is not exceptional.

## An allosemy account of manner/result polysemy

To account for manner/result polysemy, I adopt a syntactic approach to event decomposition in which acategorial roots receive their idiosyncratic interpretation only within the syntactic derivation by combining with a categorizer (Arad 2005; Borer 2005; Embick 2010). In particular, I take the relative structural position of a root to be semantically meaningful in that manner and result interpretations are linked to distinct syntactic positions within the verbal domain (Alexiadou and Lohndal 2011; Folli and Harley 2020; Hopperdietzel 2022, cf. Embick 2004; Harley 2005; Mateu and Acedo-Matellan 2012; Acedo-Matellan and Mateu 2014; and many more).

(67)

While roots that are not subject to manner/result polysemy are interpretable only in either the manner or result position, polysemous roots that are underspecified with respect to their ontological class are interpretable in both position. Therefore, I argue for an allosemy analysis of manner/result polysemy in which a single root is linked to multiple encyclopaedic entries whose distribution is determined by its morphosyntactic context (cf. Levinson 2010; Marantz 2013; Harley 2014).

(68) $[\!\![\sqrt{\mathit{underspecified}}]\!\!]$ ↔ *λ*e. manner(e) ∖ [_vP_ __ [_v’_ v (DP)]
  ↔ *λ*s. result(s) ∖ [_vP_ v [_ResP_ __ [_Res’_ Res DP]]]

In this section, I present a syntactic approach to event (de-)composition before motivating manner and result as structural notions within the verbal domain. Based on this structural account of manner and result, I develop an allosemy analysis of manner/result polysemy in which the relative syntactic position of a root governs its semantic interpretation, which is supported by suppletive verb forms in paradigm of polysemous verbs in Daakaka. Finally, I briefly discuss a diachronic perspective on the relation and formation of root allosemes based on salience, highlighting certain similarities between both cross- and intracategorial polysemy.

### A syntactic approach on event decomposition

Adopting a syntactic approach (Alexiadou et al. 2015; Folli and Harley 2020; also Ramchand 2008), I assume that event structure is built within the syntactic derivation by the layering of designated functional projections that introduce eventive and stative eventualities, including obligatory layers such as *v*P as well as optional layers such as Res(ult)P and VoiceP, into which acategorial roots are inserted (cf. templatic verb meaning in Sect. 2.1).

(69)

While the eventive categorizing head *v* introduces a dynamic eventuality *e*, the pre-categorial head Res(ult) introduces a stative eventuality *s* that is held by an entity *x*.

(70)
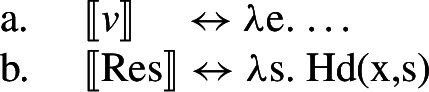

Causative semantics are interpreted configurationally via contextual allosemy when the eventive verbalizer *v* takes another eventuality as its complement (Wood 2015; Hopperdietzel 2022; cf. Higginbotham 2000; Ramchand 2008; Alexiadou et al. 2015 on telic pair formation; also Beck and Snyder 2001 on Principle R). If the eventive verbalizer merges with an individual argument, it assigns the patient/theme role to its complement.

(71) 〚*v*〛 ↔ *λ*P_<v,t>_*λ*e.∃v. Caus(e, v) ∧ P(v) ∖ ___ XP_<v,t>_
  ↔ *λ*x. *λ*e. Pat(x,e) ∖ ___ XP_<e>_

Following Alexiadou et al. (2015, 2006) and Kratzer (1996), I assume that the external argument is introduced outside of the *v*P by a separate agent-introducing Voice head, which combines with the *v*P via Event Identification (abstracting away from causer and expletive Voice for the present purpose; cf. Schäfer 2008; Martin 2020).

(72) 〚Voice〛 ↔ *λ*x*λ*e. Ag(x,e)

Consequently, verbal domain is structured by the following layers: (i) VoiceP as the locus of agentive semantics, (ii) *v*P as the locus of (causative) event semantics, and (iii) ResP as the locus of (result) state semantics.

Roots enter the syntactic derivation as acategorial modifiers that receive their idiosyncratic interpretation only in combination with a categorizing head such as *v*, *n*, or *a* (Arad 2003; Embick 2010; Alexiadou and Lohndal 2017; cf. Borer 2005). If roots combine with different categorizers, they are subject to cross-categorial polysemy, as illustrated by (instrumental) roots like $\sqrt{\mathit{hammer}}$ below. Such polysemous roots denote an entity if they attach to a nominalizer but an event if they attach to a verbalizer (Harley and Haugen 2007; Bleotu and Bloem 2020; cf. Kiparsky 1997; see Grestenberger and Kastner 2022 for an overview).

(73)

Crucially, the meaning of one variant is not entailed by the other, as indicated by the felicity of distinct instruments in verbal contexts (74a) and the lack of argument structure in nominal contexts (74b) (Arad 2003; Roy and Soare 2020).

(74)
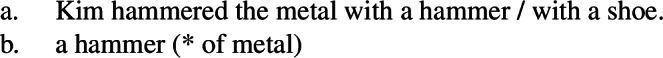

A single root may therefore be associated with multiple non-compositional interpretations which are sensitive to its local morphosyntactic environment, i.e. contextual allosemy (Marantz 1997; Harley 2014; Wood 2016; Pross 2019; Embick 2021).

(75) $[\!\![\sqrt{\mathit{h ammer}}]\!\!]$ ↔ *λ*e. hammer(e) ∖ [_vP_ ___ [_v’_ *v*]]
  ↔ *λ*x. hammer(x) ∖ [_nP_ ___ [_n’_ *n*]]

In the following, I extend the allosemy account to manner/result polysemy as a form of intra-categorial polysemy in which the interpretation of polysemous roots is sensitive to their relative syntactic position to their verbalizer (cf. Levinson 2010). To do so, I first establish manner and result as structural notions within the verbal domain.

### Manner and result as structural positions

Evidence for the different status of manner and result meaning components comes from morphosyntactic and semantic properties of two types of resultative constructions in which both meaning components are encoded by separate pre-categorized predicates: (i) resultative secondary predication (76a) (Halliday 1967; Simpson 1983; Beavers 2012) and (ii) means constructions (76b) (e.g., Dowty 1979; Sæbø 2008; Solstad 2009).

(76)
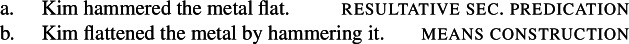

Both constructions differ significantly with respect to their internal morphosyntactic and semantic composition of the respective predicates, indicating that while result-denoting predicates merge as complements, manner-denoting predicates merge as adjuncts (Alexiadou and Lohndal 2011; Folli and Harley 2020; Hopperdietzel 2022; cf. Embick 2004; Harley 2005; Mateu and Acedo-Matellan 2012):^20^ in *resultative secondary predication*, a manner verb functions as the main predicate of the construction that combines with a pre-categorized result-denoting secondary predicate (cf. Larson 1991; Hopperdietzel 2022). Notably, the secondary predicate is in complementary distribution with the patient argument of the manner predicate. This is illustrated by resultative constructions with non-subcategorized objects below, where the patient argument of the manner verb is syntactically absent.

(77)
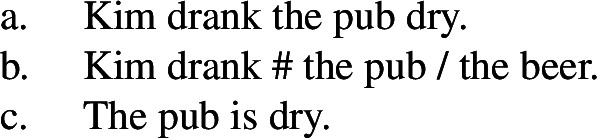

As the patient argument and the resultative secondary predicate compete for the same syntactic position, the result component is located in the complement position of *v* while the object argument is solely related to the secondary predicate where it receives the holder theta role (cf. Hoekstra 1988; Embick 2004; Kratzer 2005; but see Levin and Rappaport Hovav 1995; Williams 2012 for alternative perspectives).

(78)

The obligatory nature of the resultative secondary predicate for the causative interpretation of the complex predicate further supports its argument status as in its absence, the main predicate denotes a mono-eventive activity (cf. von Stechow 1995; Higginbotham 2000; Rothstein 2004).

(79)
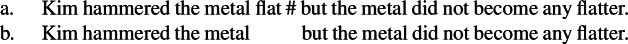

This contrasts with *means constructions* in which a causative main verb encodes the result component by itself while a secondary manner predicate specifies the underspecified causing event that is already entailed by the causative verb (Solstad 2006, 2009, 2008; cf. Dowty 1979). As such, the manner predicate functions as an event modifier and is not obligatory for the causative interpretation of the construction.

(80) Kim flattened the metal (by hammering it) # but the metal did not become any flatter.

The manner adjunct attaches at the *v*P-level as indicated by its ambiguity in periphrastic causative constructions and its felicity in the context of Voice-less unaccusative/anticausative verbs (Fodor 1970; cf. Biggs and Embick 2020). In (81), the manner adjunct *by swallowing their tongue* is ambiguous in modifying either Kim’s action that causes Robin to die or the event that caused Robin to be dead.

(81) Kim caused Robin to die by swallowing their tongue.
  (adapted from Fodor 1970)

Therefore, secondary manner predicates in means constructions merge in the modifier position of the *v*P, i.e. as sisters of *v*’, where they modify the (causing) event introduced by the verbalizing head (Sæbø 2008; Solstad 2009; Hopperdietzel 2022, 2020c; cf. Alexiadou et al. 2015 on causative from-phrases).

(82)

As a consequence, manner and result meaning components are associated with distinct syntactic positions in the verbal domain which are defined in relative configuration to the event-introducing verbalizer *v*: Manner predicates as event-modifiers merge as adjuncts in the modifier position of *v*, whereas result predicates as event-arguments merge as complements of *v* (Folli and Harley 2020; Hopperdietzel 2022).

(83)

### Manner/result polysemy as intra-categorial allosemy

In mono-morphemic verbs, roots then merge in either the manner or result position, depending on their semantic function (Embick 2004; Mateu and Acedo-Matellan 2012; Alexiadou and Anagnostopoulou 2013; Folli and Harley 2020, *inter alia*): manner roots like English $\sqrt{\mathit{hammer}}$ (86) or Daakaka $\sqrt{\mathit{ta}}$ attach as event modifiers in the modifier position *v* where they encodes the manner component of the event, introduced by *v*’ and undergo morphological (M-)merger with the categorizing head forming a manner verb (cf. Matushansky 2006).

(84)
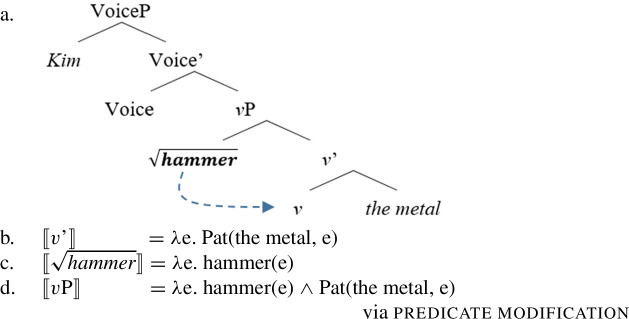

Stative result roots like English $\sqrt{\mathit{flat}}$ (87) or Daakaka $\sqrt{\mathit{mwelili}}$ instead merge as state-modifiers to a pre-categorial Res(ult)P in the complement domain of an eventive *v* head before incorporating into the verbalizer to form a causative result verb. Here, the root encodes the result component modifying the underspecified (result) state introduced by the Res(ult) head.^21^

(85)
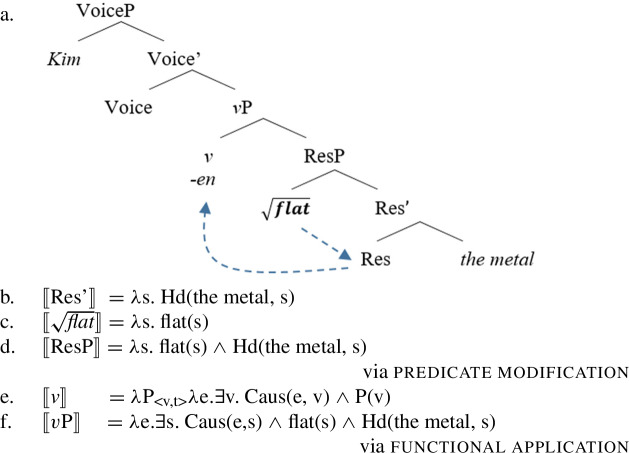

By the assumption that a single root can only be merged in a single position and a single categorizer can only categorize a single root at a time, manner/result complementary follows naturally from more general constraints on syntactic structure building (Mateu and Acedo-Matellan 2012; Folli and Harley 2020; cf. Embick 2010). The simultaneous realization of manner and result meaning thus requires one meaning component to be encoded by a pre-categorized predicate (Hopperdietzel 2022).

The ability of manner and result roots to occur in the manner and result position can therefore be motivated by their semantic type, i.e. their ontological class (cf. Rappaport Hovav and Levin 1998; Alexiadou et al. 2015; Rappaport Hovav 2017, *inter alia*): while manner roots denote sets of events which enables them to function as event modifiers, result roots denote sets of state which enables them to function as state modifiers.^22^

(86)
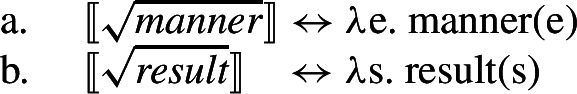

However, the existence of manner/result polysemy in contexts like English $\sqrt{\mathit{cut}}$ or Daakaka $\sqrt{\mathit{tiwi}}$ challenges such a rigid binary classification, as the same root may be able to encode a manner or result meaning component; though not simultaneously. Therefore, polysemous roots must be linked to (at least) two separate encyclopaedic entries of which one provides eventive/manner semantics and another stative/result semantics. As manner and result meaning are associated with distinct morphosyntactic positions, I propose to analyze manner/result polysemy as a case of contextual allosemy in which the relative syntactic configurationdetermines the interpretation of the root (cf. Levinson 2010; Marantz 2013; Harley 2014; Wood 2016). Therefore, polysemous $\sqrt{\mathit{cut}}$ in English denotes a ‘cutting’ action in the manner position, and a state of ‘clean separation’ in the result position.

(87) $[\!\![\sqrt{\mathit{cut}}]\!\!]$ ↔ *λ*e. cut(e)
  ∖ [_VoiceP_ Voice [_vP_ ___ [_v’_ *v* (DP)]]
  ↔ *λ*s. clean.separation(s)
  ∖ [_vP_ *v* [_ResP ____ [_Res’_ Res DP]]]

Likewise, Daakaka $\sqrt{\mathit{tiwi}}$ exhibits two allosemes that are sensitive to the syntactic position of the root, an eventive alloseme, denoting ‘a manual pressing action,’ and a stative alloseme, denoting ‘a broken state’:

(88) $[\!\![\sqrt{\mathit{tiwi}}]\!\!]$ ↔ *λ*e. press.manually(e)
  ∖ [_VoiceP_ Voice [_vP_ ___ [_v’_ *v* (DP)]]
  ↔ *λ*s. broken(s)
  ∖ [_vP_ *v* [_ResP ____[_Res’_ Res DP]]]

Consequently, when $\sqrt{\mathit{tiwi}}$ is merged in the modifier position of *v*, its eventive alloseme gets spelled-out and the resulting verb does not entail any result state, i.e. it has a monoeventive structure like other manner verbs (89).

(89)
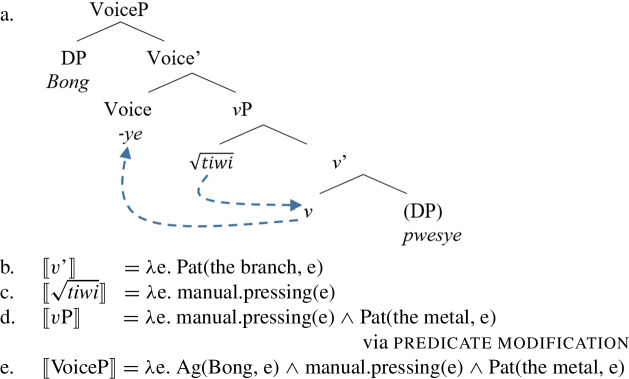

In contrast, when $\sqrt{\mathit{tiwi}}$ is attached to a stative Res(ult)P in the complement domain of *v*, its stative alloseme is spelled-out and the resulting verb does not entail a manner component, i.e. it has a bi-eventive structure like other result verbs. Due to the serializing condition, Daakaka causative verbs always occur in a means construction, i.e. in the presence of an intransitive VoiceP-sized manner adjunct (see Sect. 3.3 and fn. 13 for the adjunct status of the manner verb in RSVCs; also Hopperdietzel 2020a, 2021b for detailed analysis).

(90)
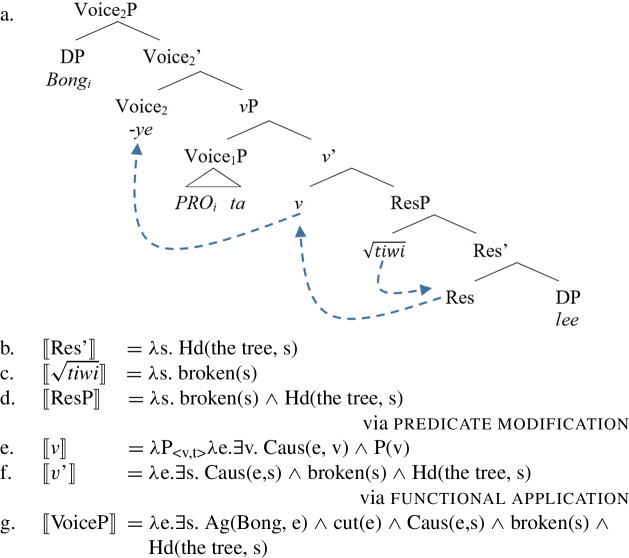

However, if roots are potentially allosemous, what restricts non-polysemous roots like $\sqrt{\mathit{hammer}}$ or $\sqrt{\mathit{flat}}$ to appear in both syntactic position? Why is manner/result polysemy not productive? Under an allosemy account, such restrictions naturally follow from the uninterpretability of roots in contexts where no alloseme is available, i.e. in the absence of an encyclopaedic entry. Only roots for which multiple encyclopaedic entries exist are therefore polysemous.

(91)
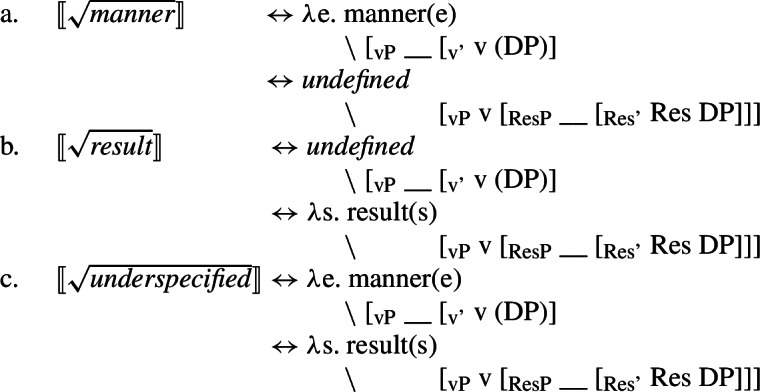

While the relative syntactic position of a root defines its interpretation, certain root classes may have additional constraints as part of their spell-out condition, with VoiceP as the relevant local domain for contextual allosemy within the verbal domain (cf. Harley and Noyer 2000; Marantz 2013; Harley 2014; Anagnostopoulou 2017): on the one hand, although Voice is usually not included in the spell-out condition of result verbs as indicated by their participation in the anticausative alternation, externally caused result roots like $\sqrt{\mathit{kill}}$ cannot form anticausative variants (92) and agentive result roots like $\sqrt{\mathit{murder}}$ even require their external argument to be agentive (93) (Levin and Rappaport Hovav 1995; Alexiadou et al. 2015; Beavers and Koontz-Garboden 2020; Ausensi et al. 2021; *inter alia*).

(92)
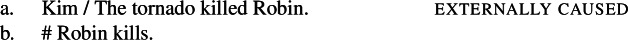

(93)
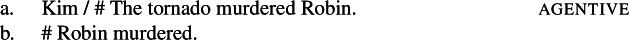

In an allosemy approach, such restrictions can be modelled as additional morphosyntactic spell-out condition to which certain classes of roots are sensitive: Externally-caused result roots require the presence of Voice, either agent or causer Voice (cf. Martin 2020), to be interpreted, whereas agentive result roots require the semantic type of Voice head as agentive (also Wood 2016).

(94)
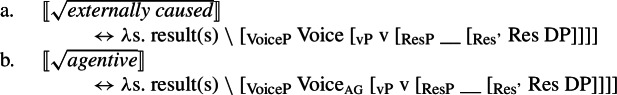

On the other hand, while most manner verbs in languages like English and Daakaka must co-occur with an agentive external argument (cf. Alexiadou 2015; Levin 2017; but see manner-of-motion verbs as discussed in fn. 3), anti-agentive constructions in languages like Mandarin, Brazilian Portuguese and Hindi/Urdu show that this constraint does not hold cross-linguistically (Carvalho 2016; Bhatt and Embick 2017; Martin et al. 2023).

(95)
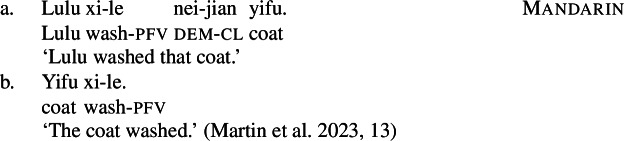

Therefore, even manner roots can differ in whether Voice is part of their spell-out condition with English-type manner roots typically including Voice and Mandarin-type manner roots typically excluding Voice (see Williams 2014; Wood 2016 for related intuitions).

(96)
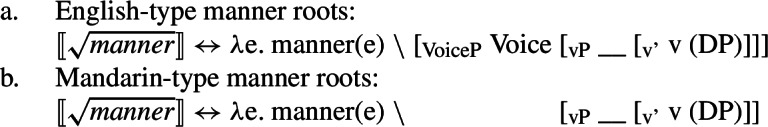

By re-interpreting certain properties of root classes as morphosyntactic spell-conditions, manner/result polysemy presents a special case of intra-categorial root allosemy that follows from two separate meanings listed for a single root that are subject to distinct spell-out condition in the verbal domain (see Levinson 2010, also Acquaviva 2009 on the nominal domain). Consequently, root meaning is sensitive to its local syntactic environment with which individual root (classes) interact in idiosyncratic ways (Arad 2003; Borer 2005; Harley 2014).

### Evidence from contextual allomorphy

As VoiceP as the spell-out domain for contextual allosemy corresponds to the spell-out domain of contextual allomorphy (Marantz 2013; Harley 2014; Wood 2015; cf. Harðarson 2021), an allosemy account makes the strong prediction that manner/result polysemy may show morphological reflexes. This prediction is borne out by suppletive verb forms in the paradigm of polysemous verbs in Daakaka. As demonstrated in Sect. 3.2.3, transitive verbs in Daakaka exhibit two types of argument structure alternations, of which transitive verb forms are marked by transitive morphology, e.g. by the synchronically productive transitive suffix *-ane* (von Prince 2015; Hopperdietzel 2020b,c): Manner verbs like *kolirane* ‘sing’ have unergative verb forms in the absence of an internal argument (97), while result verbs like *mweliliane* ‘make small’ have stative unaccusative verb forms in the absence of an external argument (98).

(97)

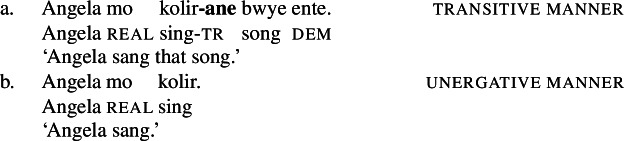

(98)
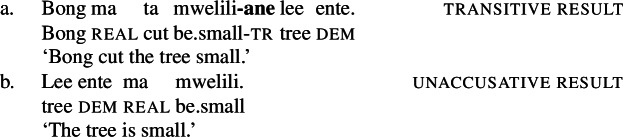

The morphological marking of the transitive alternations is thereby sensitive to the root (class) with many roots showing idiosyncratic (suppletive) transitivity morphology, such as the manner verb *te*/*ta* ‘cut’ and the result verb *wa*/*lyoo* ‘split’ (see Table 2 in Sect. 5.3 for an overview).

**Table 2:** (In)transitive verbal morphology in Daakaka idiosyncratically determined by root (class)

**Voice morphology**	**intransitive**	**transitive**	**translation**
-*ane*	*kolir*	*kolir-****ane***	‘sing’
	*mwelili*	*mwelili-****ane***	‘small’
(CVC)-V	*lung*	*lung-***u**	‘wrap’
	*min*	*min-***i**	‘drink’
*-p*	*sye***-p**	*sye*	‘slice’
	*liye***-p**	*liye*	‘take’
suppletive	**ta**	**te**	‘cut’
	***lyoo***	**wa**	‘split
suppletive + *-se*	***ves***	*vyaa***-*se***	‘kick’
	***kyes***	*ka-***se**	‘wash’
*-r/-ye*	*tiwi***-r**	*tiwi-***ye**	‘press manually’
suppletive + -*ye*	***setyup***	*tiwi***-*ye***	‘break’

(99)

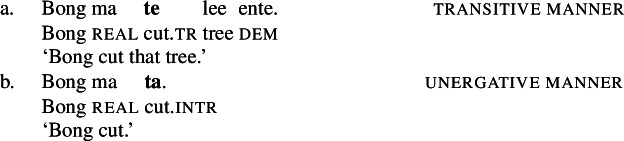

(100)
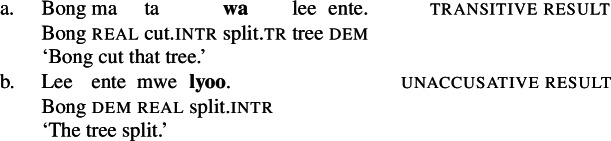

While non-polysemous verbs either have a unergative or an unaccusative intransitive verb form, polysemous verbs like *tiwiye* participate in both types of alternations in which unergative and unaccusative verb can exhibit distinct morphological marking. In the context of $\sqrt{\mathit{tiwi}}$, the unergative form is marked by the intransitive suffix -*r* (101), whereas the stative/unaccusative form *setyup* is suppletive (102).^23^

(101)

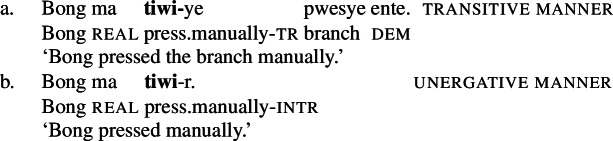

(102)
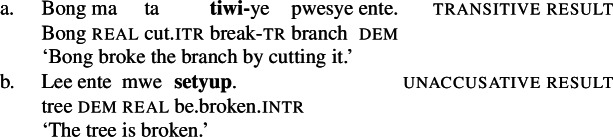

The realization of $\sqrt{\mathit{tiwi}}$ as *tiwi* is therefore sensitive to the presence of (agentive) Voice in the structure of intransitive/unergative and transitive manner, as well as transitive result verbs. If Voice is absent (or expletive; cf. Schäfer 2008), as in the intransitive/unaccusative result context, $\sqrt{\mathit{tiwi}}$ is spelled-out as its suppletive form *setyup* ‘be broken.’^24^

(103) $\sqrt{\mathit{tiwi}}$ ↔ *λ*e. press.manually(e) | /**tiwi**/
  ∖ [_VoiceP_ DP [_Voice’_ **Voice** [_vP_ __ [_v’_ *v* DP]]]
  ↔ *λ*e. press.manually(e) | /**tiwi**/
  ∖ [_VoiceP_ DP [_Voice’_ **Voice** [_vP_ __ [_v’_ *v* ]]]
  ↔ *λ*s. broken(s) | /**tiwi**/
  ∖ [_VoiceP_ DP [_Voice’_ **Voice** [_vP_ *v* [_ResP ____ [_Res’_ Res DP]]]]
  ↔ *λ*s. broken(s) | /setyup/
  ∖ [_vP_ *v* [_ResP ____ [_Res’_ Res DP]]]

Consequently, the morphological spell-out conditions for suppletive $\sqrt{\mathit{tiwi}}$ overlap with its manner and result interpretations in a fully predictable way. Daakaka therefore provides novel evidence for a syntactic implementation of manner/result polysemy along the lines of contextual allosemy, as it integrates the semantic and morphosyntactic properties of polysemous roots without any further assumptions (see Sect. 5 for a discussion of alternative accounts).

### On the prototypical relation between allosemes

Although allosemes are only indirectly related to each other, namely via a single underlying root, manner/result polysemy commonly involves an intuitive semantic relationship between the manner and result variants (see Levin and Rappaport Hovav 2013, 2014; Glass 2021; cf. Arad 2003 for a broader discussion). In particular, the manner variant of polysemous verbs often denotes an event that proto-typically leads to the result state that is entailed by its corresponding result variant, e.g. a ‘cutting’ action prototypically results in a ‘clean separation,’ a ‘set of cleaning action’ in a ‘clean’ state, etc.

(104)
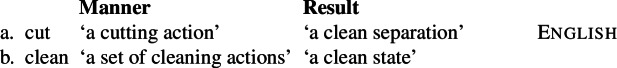

This observation also holds for Daakaka where the manner variant generally denotes a *manual* action that prototypically results in the state entailed by the result variant.

(105)
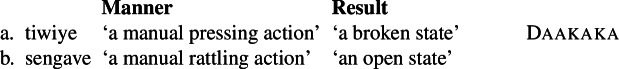

A similar observation has been made in cross-categorial polysemy where verbal and nominal allosemes may have some proto- or stereotypical relationships which seem to be sensitive to the root class (Levin 1993; Levin and Rappaport Hovav 2013; also Martin and Piñón 2020; but see Melloni 2011; Iordăchioaia et al. 2020 for a more complex picture): On the one hand, nouns based on manner roots like $\sqrt{\mathit{hammer}}$ or $\sqrt{\mathit{kick}}$ typically either denote an instrument typically involved in the event denoted by the verb or the event itself.

(106)
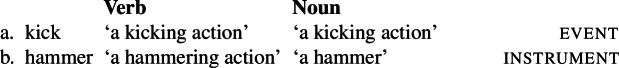

On the other hand, nouns based on result roots like $\sqrt{\mathit{flat}}$ and $\sqrt{\mathit{break}}$ typically denote an object that holds the result state or the (result) state itself.

(107)
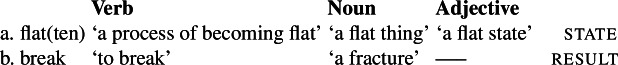

In the following, I briefly discuss such regularities by taking a diachronic perspective on alloseme formation which tentatively suggests that this process is sensitive to salience of meaning properties already associated with an existing alloseme (cf. Wechsler 2008 for a similar intuition; also Grestenberger and Kastner 2022). As etymological descriptions are unavailable for Daakaka, I primarily focus on cross- and intracatorical polysemy in English based on the examination of the earliest attested occurrence of the respective allosemes based on the *Oxford English Dictionary*.

Regarding cross-categorial polysemy, the verbal variant of the (manner) root $\sqrt{\mathit{kick}}$, which denotes the manner of an action is attested since the 14th century. The corresponding nominal variant which equally denotes a ‘kicking’ event is only attested since the 16th century.

(108) $[\!\![\sqrt{\mathit{kick}}]\!\!]$ ↔ *λ*e. kick(e) ∖ [_VoiceP_ Voice [_vP_ ___ [_v’_ *v*_<v,t>_ (DP)]]
    ∼14th century (ME)^25^
  ↔ *λ*e. kick(e) ∖ [_nP_ ___ [_n’_ n_<v,t>_ (DP)]]
    ∼16th century^26^

In contrast, the (instrumental) root $\sqrt{\mathit{hammer}}$, the nominal variant *hammer* existed since Old English referring to ‘an instrument with a hard solid head.’ The corresponding verbal variant is only attested since the 14th century and denotes the manner of action in which the entity denoted by the nominal form, i.e. ‘a hammer,’ is typically used as an instrument.

(109) $[\!\![\sqrt{\mathit{hammer}}]\!\!]$ ↔ *λ*x. hammer(x) ∖ [_nP_ ___ [_n’_ n_<e,t>_ (DP)]]
    <10th century (OE)^27^
  ↔ *λ*e. hammer(e) ∖ [_VoiceP_ Voice [_vP_ ___ [_v’_ *v*_<v,t>_ (DP)]]
    ∼14th century

The (stative) root $\sqrt{\mathit{flat}}$ is attested in its adjectival variant denoting ‘a flat state’ and in a nominal variant with the meaning of a ‘something with a flat surface’ since 14th century when it was borrowed from Old Norse. Only since the 18th century, the change-of-state verb *flatten*, denoting ‘a change into a flat state,’ is attested.

(110) $[\!\![\sqrt{\mathit{flat}}]\!\!]$ ↔ *λ*s. flat(s) ∖ [_aP_ __ [_a’_ *a*_<v,t>_ (DP)]]
    ∼14th century (ONo)^28^
   ↔ *λ*s. horizontal.plane(x) ∖ [_nP_ __ [_n’_ *n*_<e,t>_ (DP)]]
    ∼14th century
   ↔ *λ*s. flat(s) ∖ [_vP_ *v* [_ResP ____ [_Res’_ Res_<v,t>_ (DP)]]]
    ∼18th century^29^

Finally, the result root $\sqrt{\mathit{break}}$ initially formed a causative verb denoting the change into the state named by the root before the noun was established in the 14th century, denoting the result of the causative process, i.e. a fracture.

(111) $[\!\![\sqrt{\mathit{break}}]\!\!]$ ↔ *λ*s. broken(s) ∖ [_vP_ *v* [_ResP ____ [_Res’_ Res_<v,t>_ (DP)]]]
    <10th century (OE)^30^
  ↔ *λ*s. fracture(x) ∖ [_nP_ __ [_n’_ *n*_<e,t>_ (DP)]]
    ∼14th century^31^

What all four examples of allosemy have in common is that the semantics of the newly established variant is based on a salient meaning property of an already existing variant, e.g. *a hammer* is proto-typically instrument in a ‘hammering’ action and *a fracture* is a proto-typical result of a ‘breaking’ process. It therefore seems more likely for a new alloseme to lexicalize a salient meaning property of already existing allosemes.^32^

Crucially, salience also seems to be at play in intra-categorial manner/result polysemy: As highlighted by Levin and Rappaport Hovav (2013), result roots like $\sqrt{\mathit{cut}}$ denote a specific type of result state that implies the specific type of instrument/causer which facilitates the formation of a manner alloseme in which the respective instrument is entailed. The salience of an instrument/causer thus distinguishes polysemous $\sqrt{\mathit{cut}}$-type roots from non-polysemous $\sqrt{\mathit{break}}$-type roots (Bohnemeyer 2007). The etymology of *cut* tentatively supports this view, as object omission is only attested since the 16th century (cf. Sect. 3.2.3), though more systematic studies that take conative constructions into considerations are necessary.

(112) $[\!\![\sqrt{\mathit{cut}}]\!\!]$ ↔ *λ*s. clean.separation(s) ∖ [_vP_ *v* [_ResP_ ___ [_Res’_ Res DP]]]
    ∼13th century (ME?)^33^
  ↔ *λ*e. cut(e) ∖ [_VoiceP_ Voice [_vP_ ___ [_v’_ *v* (DP)]
    ∼16th century

Moreover, Glass (2021) demonstrates that result roots like $\sqrt{\mathit{lift}}$ can acquire a manner interpretation if a specific manner in which the causing event is performed becomes particularly salient, as for example in ‘weight-lifting’ routines as a (recent) form of exercising (see also Levin and Rappaport Hovav 2014 on the manner use of clean).^34^

(113) $[\!\![\sqrt{\mathit{lift}}]\!\!]$ ↔ *λ*s. raised(s) ∖ [_vP_ *v* [_ResP ____ [_Res’_ Res_<v,t>_ (DP)]]
    ∼13th cent. (OE)^35^
   ↔ *λ*e. weight-lift(e) ∖ [_VoiceP_ Voice [_vP_ ___ [_v’_ *v*_<v,t>_ (DP)]]
    not attested in OED

Consequently, manner/result polysemy is more likely to arise in domains where manner or result meanings are strongly associated with their respective counterparts which is reflected by the domains in which manner/result polysemy is commonly found cross-linguistically (cf. Segal and Landau 2012; Alexiadou and Anagnostopoulou 2013 on *clear*-type roots; Levinson 2010; Folli and Harley 2020 on creation roots; see also Martin and Piñón 2020 on denominal uses of proper and common nouns). Despite such tendencies, both intra- and cross-categorial polysemy is ultimately idiosyncratic, as alloseme formation is still unpredictable (as expected for the root categorization processes; Marantz 1997; Alexiadou 2001; Arad 2003*inter alia*; cf. Grestenberger and Kastner 2022).

## On alternative accounts

In closing, I briefly discuss three alternative analyses to manner/result polysemy, namely (i) derivation, (ii) coercion, and (iii) homophony. Although these analyses are often (implicitly) assumed to account for manner/result polysemy, I show that they face both theoretical and empirical challenges.

### Derivation

Firstly, manner/result polysemy may be treated as a derivational process, in which the manner variant is derived from the result variant, or vice versa, in the absence of overt morphology. Under a derivational account, manner/result polysemy is therefore related to other covert derivational processes that alter event structure properties of verbal predicates, e.g. the causativization of (stative) result and manner verbs (Embick 20024; Alexiadou et al. 2015, 2017; Folli and Harley 2020*inter alia*):

On the one hand, some causative variants of stative result roots like English $\sqrt{\mathit{dry}}$ for example are derived without additional causative morphology (cf. Dixon 1982 for an overview of English causativizing morphology).

(114)
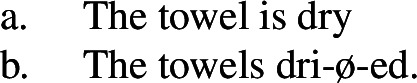

By the assumption that (lexical) causative verbs are formed by the embedding of a pre-categorial ResultP under an eventive *v* head, cases like *dry* suggest not only suggests that the spell-out of a causative *v* head is idiosyncratically determined by the root (via contextual allomorphy; Alexiadou 2015; Wood 2015; Hopperdietzel 2021a, cf. Moskal 2015; Bobaljik 2012; Embick 2010), but that it can remain silent.

(115) *v* ↔ -en  ∖ [_vP_ ___ ResP]] {$\sqrt{\mathit{flat}}$, $\sqrt{\mathit{dark}}$, …
  ↔ ø ∖ [_vP_ ___ ResP]] {$\sqrt{\mathit{dry}}$, $\sqrt{\mathit{clean}}$, …

Semantically, the causative verbalizer introduces an underspecified event that is in a causative relation with the (result) state specified by the root within the ResultP. Therefore, silent derivational morphology may derive eventive/causative verb forms from stative (result) roots adding a causing event to the event structure.

(116)
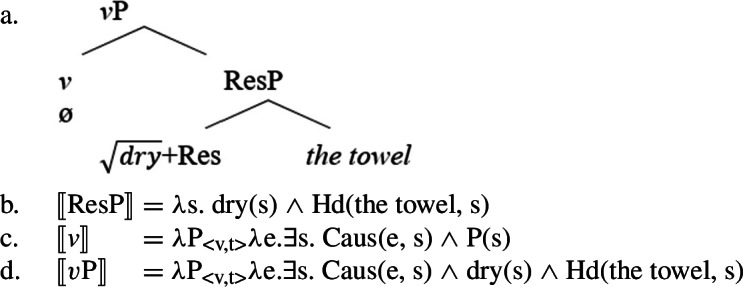

On the other hand, manner verbs like French *balayer* ‘to sweep’ can receive a causative interpretation again in the absence of overt derviational morphology (Alexiadou et al. 2017; also Hopperdietzel 2020c on Samoan; Anagnostopoulou 2017 on Greek). Unlike its manner variant, causativized *balayer* can combine with (inanimate) causers like *vent* ‘wind’ and a denial of result becomes infelicitous.

(117)
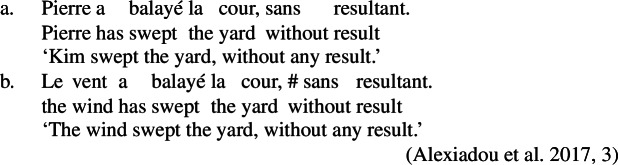

However, although not causative result and causative manner verbs denote causative eventualities, they crucially differ in that the latter leaves the nature of the result state underspecified. Instead, the result state is interpreted as the state that is conventionally associated with the successful (action) event denoted by the manner verb. Causative manner verbs thus maintain their manner component which restricts the choice of (inanimate) causer subjects to the ones that are compatible with it, as illustrated by the infelicity of *pluie* ‘rain’ below.

(118)
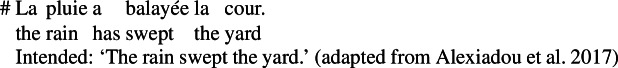

Causative manner verbs are formed by a manner verb embedding a silent ResultP which introduces an underspecified result state that is in a causative relation with the event introduced by the verbalizer *v*. (cf. Embick 2009).^36^ This (causing) event is then identified by a manner root like $\sqrt{\mathit{balayer}}$.

(119)
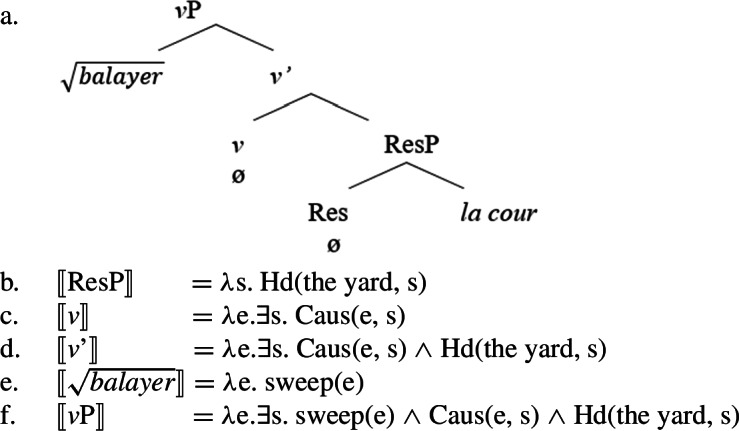

Consequently, causative variants may be derived from both eventive manner and stative result roots by silent causative or silent resultative morphology. As silent word formation is therefore possible, manner/result polysemy could be argued to follow from zero derivation in which a silent morpheme derives the manner interpretation from the result interpretation (120), or vice versa (121).

(120)
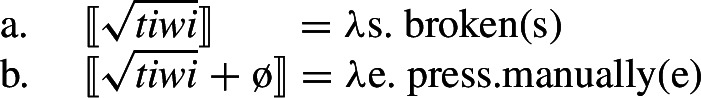

(121)
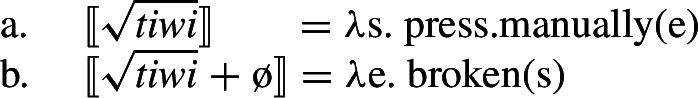

However, since manner and result variants do not entail the semantics of their counterparts, an operation that describes the derivational step from manner to result interpretation, or vice versa, would violate the monotonocity constraint on word formation (Rappaport Hovav and Levin 1998; Koontz-Garboden 2007, 2012).

(122) **The principle of monotonic composition (PMC)**
  Word formation operations do not remove operators from lexical semantic representations.
    (Koontz-Garboden 2012, 143)

According to the PMC, word formation is strictly monotonic in that meaning may be added to a semantic representation but not removed. While the causativization of manner and result verbs is monotonic in adding a (causing) event or a (result) state to the root meaning, manner/result polysemy is non-monotonic as the original root meaning needs to be replaced. In contrast to derivational accounts, an allosemy account reflects the non-compositional nature of manner/result polysemy and obeys the PMC, as both manner and result variants are independently derived and do not entail each other.

### Coercion

Secondly, manner/result polysemy may be analysed as an instance of pragmatic coercion in which the interpretation of a lexeme is shifted to repair a semantic mismatch in the semantic interpretation of the syntactic structure (cf. de Swart 1998; Zucchi 1998; Koontz-Garboden 2007). A coercion analysis has been applied to event structure alternations, such as the stative/anticausative alternation in the Tongan (Koontz-Garboden 2007). In this language, stative verbs receive an anticausative interpretation when they occur in the context of syntactic material that operates on eventive predicates, such as aspectual heads, or adverbials. This is illustrated in (123) where the stative verb *loloa* ‘be long’ gets a dynamic interpretation ‘get long’ in the context of the eventive modifier *vave* ‘fast.’

(123)
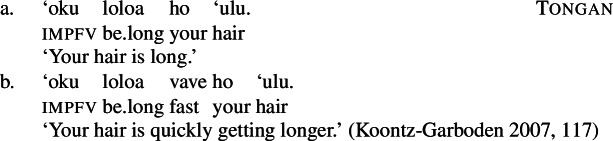

The application of a coercion analysis to manner/result polysemy seems promising at first glance, as one variant of polysemous verbs is restricted to specific morphosyntactic environments (e.g., Roßdeutscher and Kamp 2010), e.g., the manner variant of English *cut*. Consequently, it could be argued that the morphosyntactic contexts shifts the meaning of the root to the appropriate semantic type, i.e. state or event.

However, manner/result polysemy is not necessarily sensitive to its morphosyntactic context. The polysemous root $\sqrt{\mathit{clean}}$, for example, can be interpreted as a manner or as a result root in the same morphosyntactic context (Levin and Rappaport Hovav 2014). In the example below, *clean* either refers to the manner of the cleaning routine performed by the hygienist (124a) or the teeth coming into a clean state as a result of the treatment the hygienist, as indicated by the entailment of a result state only in the latter (124b). This ambiguity therefore suggests that manner/result polysemy may be available independently without the presence of coercive structure.

(124)
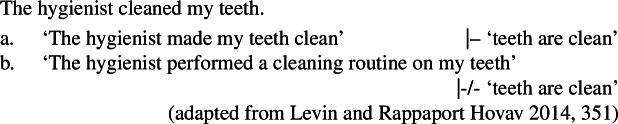

Moreover, coercion as a pragmatic operation is expected to obey the PMC, i.e. meaning may be added but not removed (Michaelis 2004; Koontz-Garboden 2012, 2007). Stative verbs in Tongan, for example, are therefore obligatorily coerced into anticausative but never into activity verbs, as the latter would a require a cancellation of the state variable. As a result, coercion accounts of manner/result polysemy run into the same problems as derivational accounts as they violate the PMC, since manner and result meaning components of polysemous verbs are in complementary distribution.

(125) 〚*loloa*〛 = *λ*s. long(s) →_coercion_ *λ*e.∃s. Caus(e,s) ∧ long(s)
  # →_coercion_ *λ*e. long(e)

Another counterargument comes from suppletive forms in the paradigm of polysemous verbs in Daakaka which exhibit intransitive unergative manner (126b) and potenitally suppletive unaccusative result forms (127b) (see Sect. 4.4).

(126)

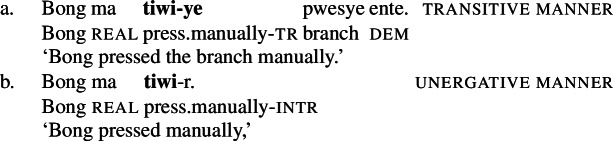

(127)
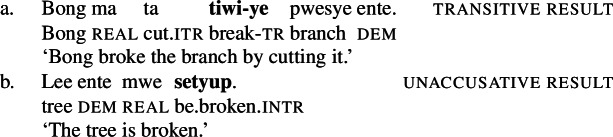

While the distribution of suppletive forms follows naturally from an allosemy account as contextual root allomorphy is expected to be sensitive to the presence or absence of (agentive) Voice in the context of transitive verbs, a distinct morphological marking is rather unexpected for a post-syntactic pragmatic repair mechanism like coercion. In particular, a coercion analysis implies a designated morphophonological encyclopaedic entry for a constituent that is void of semantic interpretation; an assumption that seems undesirable on independent grounds. Instead, we might expect regular/default morphological marking, as for example plural *-s* in mass/count coercion in English (cf. Wiese and Maling 2005).

(128) Kim drank two coffee**-s**.

Consequently, a coercion analysis not only faces theoretical challenges as it violates PMC but also struggles to account for morphological reflexes of manner/result polysemy, as observed in suppletive paradigm in Daakaka.

### Homophony

Finally, manner/result polysemy may be analysed as a case of mere homophony, i.e. manner and result variants of polysemous verbs like Daakaka *tiwiye* are actually formed by two distinct underlying roots which happen to have the same morphophonolgical realization and are independently stored in the encyclopaedia (as suggested for cases of cross-categorial polsemy such as hand by Beavers and Koontz-Garboden 2020).

(129)
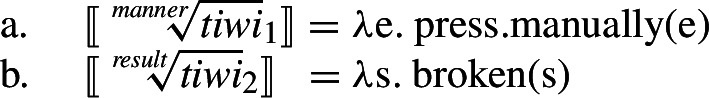

Manner and result variants would be equivalent to cases of accidental homophony of diachronically independent roots, such as *kosten* ‘to taste, to cost’ below. Manner/result polysemy would then be interpreted as a special case homophony in which the meaning of certain roots split into two independent entries at some diachronic stage (cf. Grestenberger and Kastner 2022 on root augmentation).

(130)
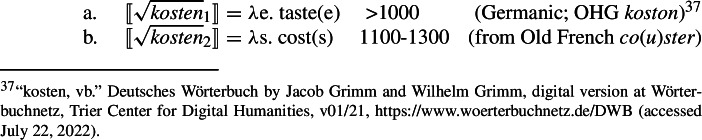

While a homophony account to manner/result polysemy can capture the semantic and morphosyntactic challenges for derivational and coercion approaches, it loses a synchronically active link between the manner and the result variant of polysemous verbs. A morphological counterargument comes from Daakaka where Voice morphology is idiosyncratically determined by the root (class) (von Prince 2015; Hopperdietzel 2020b,c).

Building on Aronoff (1976, 2007), Harley (2014) argues that a single underlying root can be identified based on idiosyncratic morphological realizations which are constant across morphosyntactic environments and semantic interpretations. In English for example, verbs like *perceive, conceive*, or *receive* are derived from a single (bound) root $\sqrt{\mathit{ceive}}$ which is meaningless outside of its morphosyntactic contexts but is subject to the same allomorphy rules, i.e. its realization as the allomorph *-cept* in the context of the nominalizer -*ion*.

(131) *-ceive* ∼ *-cept* + *ion*
  perception, conception, reception, etc. (Harley 2014, 241)

In the same way, idiosyncratic Voice morphology in Daakaka is shared between manner and result variants of polysemous verbs; not only is $\sqrt{\mathit{tiwi}}$ is spelled as *tiwi-* in the context of Voice in both variants (121) but it also takes the same allomorph of transitive Voice independent of its interpretation (125) (cf. Table 2).

(132) Voice_tr_ ↔ -ye ∖ {$\sqrt{\mathit{tiwi}}$
  ↔ -V ∖ {$\sqrt{\mathit{lung}}$, $\sqrt{\mathit{min}}$, …
  ↔ *suppletive* ∖ {$\sqrt{\mathit{ta}}$, $\sqrt{\mathit{wa}}$, …
  ↔ -ane elsewhere

Suppletive Voice morphology in Daakaka therefore indicates that manner and result variants are derived from a single underlying root. While these morphological facts are rather unexpected under a homophony account, they are naturally accounted for under an allosemy account. As a diachronic explanation still seems to be feasible however, discriminating evidence may ultimately come from psycho- and neurolinguistics research, which revealed that cross-categorial polysemy differs from homophony in that only polysemous word forms share some synchronically active common core (Frazier and Rayner 1990; Pylkkänen et al. 2006; Klepousniotou et al. 2012). Whether these results carry over to intra-categorial manner/result polysemy is an open question for future research.

## Conclusion

In this paper, I approached the phenomenon of manner/result polysemy from the perspective of the Oceanic language Daakaka. The examination of the morphosyntactic and semantic properties of the polysemous verb *tiwiye* ‘press manually, broken’ provided novel cross-linguistic evidence for the complementary distribution of manner and result meaning in polysemous verbs, as the manner component appears to drop in the result variant, and vice versa. Therefore, manner/result polysemy in Daakaka does not represent a counterargument to the hypothesis of manner/result complementarity as a potentially universal lexicalization principle.

However, the observation that a single root can either specify the manner or result component of an event challenges a binary classification of roots into ontological classes, as commonly proposed in the literature. Based on the morphosyntactic properties of manner/result roots in Daakaka, I developed an allosemy account of manner/result polysemy in which the relative morphosyntactic position of acategorial roots to an event-introducing verbalizer *v* is semantically meaningful and determines their interpretation. Considering the properties of manner and result secondary predicates in resultative constructions, I motivated that the manner interpretation is associated with the modifier position of *v*, whereas the result interpretation is associated with the complement domain of *v*. Consequently, polysemous roots have multiple encyclopaedic entries that exhibit spell-out conditions that are sensitive to the morphosyntactic position the root appears in.

Although allosemes of a single root are therefore only indirectly related, I highlighted that their semantic relationship is often intuitive and sometimes even predictable, as in the case of the ‘routine’ manner interpretation. To account for this observation, I sketched out a diachronic perspective taking into consideration that allosemes enter the encyclopaedia at different diachronic stages. Thereby, I suggested that the salience of certain semantic properties of existing root meaning influences the interpretation that the root acquires if it is inserted into a new morphosyntactic configuration by speakers. Further investigation of etymological development of root meaning, e.g. with the help of historical corpora, are needed to verify this hypothesis.

Finally, I discussed alternative approaches to manner/result polysemy, including derivation, coercion, and homophony, which all struggle to account for its morphosyntactic and semantic properties on both theoretical and empirical grounds. In particular, I showed that derivational and coercion analyses violate the monotonocity principle of word formation, as manner/result polysemy is essential non-monotonic. Moreover, coercion and homophony analyses do not predict suppletive morphological forms in the paradigm of polysemous verbs in Daakaka. As spell-out conditions for contextual allomorphy and contextual allosemy correspond to each other, an allosemy account naturally predicts the observed pattern.

More generally, an allosemy perspective on root meaning contributes to our understanding of the underlying nature of root classes, as (some of) their properties can be understood as spell-out conditions on their interpretation, as illustrated for result verbs in (128).

(133)
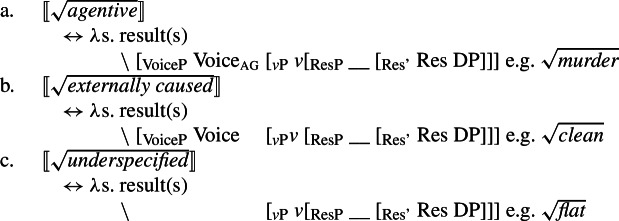

Therefore, this account allows for an integration of various underlying morphosyntactic and semantic properties of root classes into a more general theory of root categorization, which makes further predictions about potential cross-linguistic variation. On the one hand, given locality constraints on contextual root allosemy, the account restricts the local domain of root class-defining projections to the VoiceP phase, which appears to be borne out by the empirical picture (Marantz 2013; Anagnostopoulou and Samioti 2014; Harley 2014; but see Panagiotidis 2014 for a more nuanced view).

(134)

Cross-linguistic variation of various properties of root classes may then follow from language-specific spell-out conditions that are sensitive to the presence of functional structure in the respective domain, as suggested for the contrast between manner verbs in Mandarin and English of which only the latter are sensitive to Voice (see also Williams 2014).

(135)
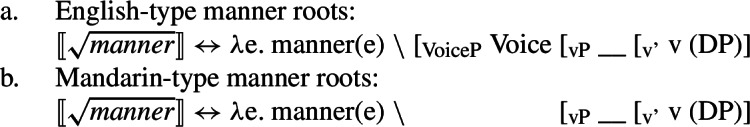

On the other hand, two allosemes of the same root are not expected to occur in free variation, as it predicts a one-to-one mapping between morphosyntactic position and encyclopaedic entry, due to the blocking by existing allosemes (cf. Wechsler 2008 for similar intuitions on English *growth*). In the context of manner/result polysemy, I have argued that underspecified polysemous roots merge in distinct morphosyntactic position with the stative (result) interpretation following from the presence of a pre-categorial Result head.

(136) $[\!\![\sqrt{\mathit{underspecified}}]\!\!]$ ↔ *λ*e. manner(e) ∖ [_vP_ __ [_v’_ v (DP)]
  ↔ *λ*s. result(s) ∖ [_vP_ v [_ResP_ __ [_Res’_ Res DP]]]

In addition, root meaning may also be influenced by the semantic-type of the categorizer itself, as in combination of roots with entity- and event-denoting nominalizers, as with German *Wurf* ‘(a) throw’ vs. *Würfel* ‘(a) dice’ (∼ ‘a specific thing that is thrown in games’; see fn. 35).

(137) $[\!\![\sqrt{\mathit{werf}}]\!\!]$ ↔ *λ*e. throw(e) | /Wurf/ ∖ [_nP_ __ [_n’_ n_<v,t>_]]
  ↔ *λ*x. dice(x) | /Würfel/ ∖ [_nP_ __ [_n’_ n_<e,t>_]]

Yet, root polysemy is not restricted to semantic-types, as spell-out conditions are sometimes sensitive to more fine-grained semantic features, such as [+/− agentive] on Voice in (133) (Acquaviva 2009; cf. Starke 2009 et seq. for a nanosyntax perspective). The challenge is therefore to identify the morphosyntactic inventory that is relevant for root polysemy in any given language, ultimately returning to the broader question about the granularity of syntactic decomposition of root meaning and the structure of the encyclopaedia.

## Footnotes

1 In this paper, I adopt the view that BECOME = CAUSE, i.e. intransitive anticausative verbs differ from transitive causative verbs primarily in the absence of a causer argument (Kratzer 2005; Alexiadou et al. 2006). However, the assumption of a separate BECOME event in change-of-state predicates in the tradition of Rappaport Hovav and Levin (1998) and Dowty (1979) does not affect the general argumentation.

2 As the results of this study are in line with the hypothesis of manner/result complementarity, I will not further address potential counterexamples, as they are not directly relevant for the argumentation.

3 Note that these criteria are not sufficient for manner/result complementarity, as in English, only (but not all) manner verbs can occur in the conative construction and only (but not all) result verbs can occur in the anticausative constructions (see Sect. 3 for further diagnostics and discussion of cross-linguistic variation).

4 Not all manner verbs require their sole argument to be agentive. In English, manner-of-motion verbs like *roll* or *bounce* exhibit mono-eventive properties in the absence of an agent (Schäfer 2012; Rappaport Hovav 2014). Moreover, languages like Mandarin allow manner verbs to appear in the anti-agentive construction in which the agent argument is structurally omitted in the context of monoeventive but telic manner verbs (Martin et al. 2023; cf. Bhatt and Embick 2017 on Hindi/Urdu; Carvalho 2016 on Brazilian Portuguese).

5 Again, this diagnostic may not be sufficient in languages that exhibit defeasible causative (Martin and Demirdache 2020). In Indonesian for example, a denial of result state of causative verbs like *mentutup* ‘close’ is felicitous in agentive, but not in non-agentive contexts (Sato 2020).

6 *Result* roots differ from *stative* roots in that they may not allow for restitutive readings of repetitive modifiers like *again* for all speakers (Beavers et al. 2021 for a cross-linguistic picture; cf. von Stechow 1996); yet for some speakers, including one reviewer, this reading seems available (also Beavers and Koontz-Garboden 2020, fn. 21).

Beavers and Koontz-Garboden (2012, 2020) therefore suggest that ‘result’ roots are semantically more complex in that they entail an existentially-bound causing event (or “change” event, in their terms). Embick (2009) instead proposes that result roots are event modifiers, similar to manner roots, but require the presence of an (underspecified) result state (also Alexiadou et al. 2017; Anagnostopoulou 2017). Recently, Yu et al. (2023) argue that result roots are eventive and entail the change into a result state which is specified by the root. Whether the interpretational constraints on ‘result’ roots is attributed to the semantic content of the root itself or to configurational restrictions on its interpretation is orthogonal to the argumentation in this paper.

7 Another verb that shows manner/result polysemy is *sengave* ‘rattle manually, open’ which patterns like *tiwiye*.

Moreover, the verb *guone* ‘perform manual cleaning action, clean’ denotes a set of manual actions that have the purpose of ‘cleaning’ in its manner variant (e.g., preparing Kava roots for a drink, removing ash from a shirt, or dust from a bench), though without entailing a result state (similar to German manner verb *putzen* ‘clean’ and the manner use of English polysemous clean; Levin and Rappaport Hovav 2014; Alexiadou et al. 2017). In its result variant, it denotes the change into a ‘clean’ state.

However, the synchronic status of *guone* is unclear. While some speakers still use it (though rarely), most speakers do not use it actively (cf. Krifka 2013 for data on the closely related language Daakie where the cognate verb *gone* exists which may influence bilingual speakers).

8 As mentioned in fn. 4 and fn. 5, certain manner/result diagnostics may not be cross-linguistic applicable due to independent language and verb-class specific properties, e.g. anti-agentivity, non-culmination, etc. which is why I focus on a set of diagnostics that are applicable to the Daakaka data.

9 The properties of certain result states may restrict the compatibility of result verbs and instruments. In English for example, *cut*-verbs lexicalize a result state that presupposes that the object got in contact with a sharped-edged object (cf. Guerssel et al. 1985; Bohnemeyer 2007). While this object is typically an instrument used by an agent (cf. Rissman et al. 2015), this presupposition is also active in agent-less anticausative contexts where the causing event-participant may be expressed as a locational modifier (cf. Levin and Rappaport Hovav 2013).

10 Mokrane, Mourad. 2016. Bend. In *Noun Project*. URL: https://thenounproject.com/icon/bend-421208/. Accessed 13/06/2023.

11 Couqet, Adrien. 2020. Break. In *Noun Project*. URL: https://thenounproject.com/icon/break-3968613/. Accessed 13/06/2023.

12 This observation is sometimes linked to the argument-per-subevent condition, namely that each sub-event needs to be related to (at least) one syntactically-represented argument XP (Levin and Rappaport Hovav 2001). Accordingly, mono-eventive manner verbs facilitate object omission, whereas bi-eventive result verbs do not.

13 Two reviewers wondered whether the availability of a denial of result in the independent use of *tiwiye* may be related to the phenomenon of non-culminating causatives with agentive subjects (cf. Martin and Demirdache 2020). Yet, unlike non-culminating causatives, independent *tiwiye* additionally entails a manner component that is not present in serializing contexts. This indicates that independent *tiwiye* is not an instance of non-culmination. In addition, we expect non-culminating reading to disappear in the context of causer subjects. In Daakaka, causer subjects often occur in paratactic constructions or in periphrastic causatives. However, some causers can also combine with a small class of manner verbs whose (abstract) manner component seem somewhat compatible with a natural force, including *kase* ‘wash,’ *te* ‘cut,’ *ane* ‘eat,’ and *tiwiye* ‘press manually’ (i). These combinations are highly restricted as only a single type of causer can combine with a respective verb, e.g. *byata* ‘lightning’ only combines *te* but not with *tiwiye*. Some speakers also mention a personification flavour in such contexts.

Significantly, in combination with a causer subject, these manner verbs gain some result properties in that they now reject both object drop (iia) and a denial of a result becomes infelicitous (iib).

This pattern resembles *optionally causative manner verbs* in languages like French, i.e. manner verbs that optionally combine with a (silent) result state in the context of causer subjects, e.g. *balayer* ‘sweep’; a phenomenon distinct from both non-culmination and polysemy (see Sect. 5.1; Alexiadou et al. 2017; Anagnostopoulou 2017).

14 Like *ta* ‘cut,’ Daakaka *sye* ‘slice’ and *tyo* ‘rip’ differ from their English translations: while in English, *slice* and *rip* are result verbs entailing a result state, this is not the case in Daakaka where such verbs resemble other manner verbs, i.e. require an instrument, allow object omission, and denial of result. *Ta* and *sye* primarily vary in the type of ‘cutting’ action performed by the agent: *ta* usually denotes a more uncontrolled ‘hacking’ action, typically but not necessarily performed with a heavy blade; *sye* instead refers to a more controlled action, typically but not necessarily performed with a slender blade (also von Prince 2017). Crucially, both verbs do not entail any change-of-state. *Tyo* denotes a ‘pulling’ action performed on two sides of an object without entailing that the object actually gets torn. These subtle differences in meaning highlight the importance of a careful examination of the lexical semantic properties in cross-linguistic research.

15 While the manner component in Daakaka RSVCs can be realized by multiple manner verbs, the result component can only be realized by a single causative result verb, even if one of the result verbs further specifies the result state introduced by the other result verb (i), i.e. RSVCs cannot form weak resultatives or (unselected) resultatives with result verbs in the initial position (cf. *She tore him free*; Yu et al. 2023). Instead, a meaning similar to weak resultatives must be realized by an adverbial RSVCs in sentence-final position, as in (53a).

16 That the attachment of the transitive suffix derives causative verbs from stative property concept verbs is supported by data from closely related languages North Ambrym (i) and Vatlongos (South-East Ambrym), which do not exhibit a serializing condition on causatives (Franjieh 2012; Ridge 2019).

17 Additional cross-linguistic support for the verbal status of causative verbs comes from related Oceanic languages such as Vurës (North Vanuatu, Oceanic; Krauße 2022), Ahamb (Central Vanuatu, Oceanic; Rangelov 2020) and Paamese (Central Vanuatu, Oceanic; Crowley 2002, cf. Bradshaw 1982) which exhibit a productive way to derive intransitive stative/anticausative verbs from obligatorily serialized transitive causative verbs by the application of a reflex of the stativizing/anticausativizing prefix *m*V- (cf. Evans 2003), a process lost in Daakaka.

18 By assuming that transitive morphology is the spell-out of two checked nominal licensing features on the agent-introducing Voice head (Hopperdietzel 2020b; cf. Deal 2010; Sigurðsson 2012; Nie 2017), its occurrence on the lower non-initial predicate is only expected in the absence of an intervening manner verb (ib) (Hopperdietzel 2021b, 2020c; cf. Baker 1985; Arregi and Pietraszko 2020). If the manner verb were the main predicate, transitive morphology would be expected to occur only on the manner (ia) (see Sect. 4.2 for the syntax of RSVCs).

Yet, Daakaka RSVCs differ from English means constructions in that the manner predicate is obligatory intransitive, despite the presence of a Voice head. In English instead, the means adjunct cannot only be transitive (iia) but may also contain a separate object argument (iib/iic) (cf. Truswell 2007).

The intransitivity constraint follows from the absence of a second nominal licensing feature on non-finite VoiceP-adjunct in Daakaka means constructions (Hopperdietzel 2022, 2021b; cf. Legate 2014; Nie 2020). In the absence of passive Voice in Daakaka (von Prince 2015), Voice solely licenses an external argument PRO (with patient argument being omitted), and is spelled-out as intransitive morphology, as in (ib). As object omission is only available for manner verbs, the limited licensing capacity within the means adjunct explains the absence of result verbs in the initial position of Daakaka RSVCs, although causative means adjuncts are felicitous in other languages (iic). However, the preposition *by* functions as an additional licenser in English *means* adjuncts, allowing transitive verbs to introduce a (distinct) object argument (cf. Alexiadou 2013).

19 See Hopperdietzel (2021b, 2020c) on the serializing condition as a language specific constraint at the syntax-semantics interface that rules out existential closure over event variables that are not realized morpho-phonologically (cf. Zimmermann 2007; Backlawski Jr. 2018; for similar observations in unrelated languages).

20 The structural analysis of manner and result will be motivated based on English, which, as a satellite-framed language, straightforwardly allows both manner and result to be realized by pre-categorized XPs. This contrasts with Daakaka which, as a verb-framed language, requires the result component to be incorporated into the main predicate (Talmy 2000; Folli and Harley 2020; Hopperdietzel 2022, 2020c).

21 As noted in fn. 6, $\sqrt{\mathit{break}}$-type roots are subject to a recent debate about the status of result roots that entail change-of-state semantics but unlike $\sqrt{\mathit{flat}}$-type roots lack a simple stative form; as for example diagnosed by the absence of a restitutive reading. To account for this observation, Beavers and Koontz-Garboden (2020) argue that $\sqrt{\mathit{break}}$-type roots are result roots that inherently denote result states, i.e. lexically entail change-of-state semantics. In contrast, Embick (2009) suggests that $\sqrt{\mathit{break}}$-type are manner roots, i.e. event modifiers, that require the presence of an underspecified result (see also Yu et al. 2023 for arguments that result roots are eventive). As both accounts are in principle compatible with my analysis, I do not go into detail here.

22 A reviewer wonders whether manner roots are always eventive as ‘instrumental’ roots like $\sqrt{\mathit{hammer}}$ (ia) as well as common and proper nouns like *butcher* (ib) and *Obama* (ic) may encode the manner component of a verb.

With respect to instrumental roots, it has been argued that the meaning of the verbal variant is independent of its nominal variant, as the instrument is not entailed by the verb but only an action of which the instrument is prototypically a part of (see Sect. 4.1 for arguments and discussion; cf. Bleotu and Bloem 2020). Likewise, (manner) verbs based on roots forming common and proper nouns do not entail the individual(s) in their verbal use, i.e. in (ib) Kim does not need to be a butcher, and in (ic) Bernie Sanders is not Barack Obama, but again refer to a proto- or stereo-typical behaviour associated with the common or proper noun (see Martin and Piñón 2020 for a detailed discussion and a more complex picture). It can therefore be argued that such verbs are actually not denominal but derived from acategorial roots that independently derive nominal and verbal meanings via contextual allosemy in the context of the respective categorizer (see example (74) for an analysis of $\sqrt{\mathit{hammer}}$). As will be discussed in Sect. 4.5, such prototypicality is also commonly observed in the context of manner/result polysemy where the manner variant denotes an event that prototypically causes the state entailed by the result variant. In brief, I propose that the salience of an event participant or event component facilitates the formation of new allosemes of a root. For example, the salience of the action which is prototypically performed with the individual denoted by the nominal alloseme of the root $\sqrt{\mathit{hammer}}$ allowed the formation of a verbal alloseme which denotes the manner of that action (cf. Glass 2021 on the relevance of contextual salience).

23 However, intransitive verb forms of polysemous verbs are not necessarily formed by suppletion, e.g. *sengave* ‘rattle manually, open’ where unergative manner (ia) and unaccusative result verbs (ia) exhibit the same form.

24 A reviewer wonders whether *setyup* and *tiwiye* are suppletive verb forms of the same root or rather two independent verbs with a similar meaning. Evidence for a suppletion analysis comes from change-of-state verbs which come in pairs of intransitive stative/anticausative and transitive causative forms. While for many verbs the causative form is transparently marked by the transitive Voice marker *–ane* (ia), some verbs show root suppletion (ib).

Notably, Daakaka also exhibits Voice-conditioned suppletion in other context, e.g. in the alternation of intransitive unergative and transitive forms (see Table 2 for an overview of Daakaka transitive Voice morphology that is sensitive to root class; also von Prince 2015).

The observation that suppletive (in)transitive Voice-marking is a general property of verbal predicates in Daakaka thus strongly suggests that *setyup* and *tiwiye* are indeed suppletive forms of a single underlying root (cf. Borer 2014; Harley 2014 for further discussion).

25 “kick, v.1.” OED Online. June 2022. Oxford University Press. https://www.oed.com/view/Entry/103264 (accessed July 22, 2022).

26 “kick, n.1.” OED Online. June 2022. Oxford University Press. https://www.oed.com/view/Entry/103261 (accessed July 22, 2022).

27 “hammer, n.1.” OED Online. June 2022. Oxford University Press. https://www.oed.com/view/Entry/83743 (accessed July 22, 2022).

28 “flat, adj., adv., and n.3.” OED Online. June 2022. Oxford University Press. https://www.oed.com/view/Entry/71171 (accessed July 22, 2022).

29 “flatten, v.” OED Online. June 2022. Oxford University Press. https://www.oed.com/view/Entry/71213 (accessed July 22, 2022).

30 “break, v..” OED Online. June 2022. Oxford University Press. https://www.oed.com/view/Entry/22915 (accessed July 22, 2022).

31 “break, n.1.” OED Online. June 2022. Oxford University Press. https://www.oed.com/view/Entry/22913 (accessed July 22, 2022).

32 Synchronically, the salience between two allosemes may be lost. In Hebrew for example, the root $\sqrt{\mathit{kbf}}$ ‘press’ forms nouns like *kviě* ‘highway’ and *kavuě* ‘pickles’ which historically share some kind of ‘pressing’ but whose synchronic relation is opaque (Aronoff 2007; Harley 2014; Grestenberger and Kastner 2022).

33 “cut, v.” OED Online. June 2022. Oxford University Press. https://www.oed.com/view/Entry/46341 (accessed July 22, 2022).

34 Intra- and cross-categorial allosemy may also interact in the formation of polysemous verbs, as suggested by the etymology of the German polysemous verb *würfeln* ‘roll dice, cut into dices.’

Initially, the noun *Würfel* ‘dice’ was derived from the root $\sqrt{\mathit{werf}}$ ‘throw’ by the OHG instrumental suffix * -*il* in the 10th century. The meaning thus reflects the non-compositional nature of cross-categorial polysemy, as *Würfel* does not entail an event but simply denotes an ‘entity that is thrown in games.’ From 16th century on, the verb *würfeln* ‘to roll dice’ is attested, denoting an action, in which the instrumental noun is typically but not necessarily used. Due to semantic bleaching of *Würfel* from ‘dice’ to ‘cube’ in the 16th century, compounds like *Spielwürfel* ‘play cubes’ are attested. Based on the semantically bleached noun, the result verb *würfeln* ‘to dice’ is found since the 18th century, denoting a prototypical process of producing small cubes in cooking routines (cf. “würfeln, vb.” Deutsches Wörterbuch von Jacob Grimm und Wilhelm Grimm, digitalized version in Wörterbuchnetz of the Trier Center for Digital Humanities, v01/21, https://www.woerterbuchnetz.de/DWB; accessed July 22, 2022).

(ii) $[\!\![\sqrt{\mathit{werf}}]\!\!]$ ↔ ‘to throw’ | /werfen/ ∖ [_vP_ __ [_v’_ *v* PathP]] <8th cent. (OHG)
  ↔ * ‘a die’ | /Würfel/ ∖ [_nP_ __ [_n’_ *n*_<e,t>_ (DP)]] ∼10th century
  ↔ ‘to play with dice’ | /würfeln/ ∖ [_vP_ __ [_v’_ *v* (DP)]] ∼16th century
  ↔ ‘a cube’ | /Würfel/ ∖ [_vP_ __ [_v’_ *n*_<e,t>_ (DP)]] ∼16th century
  ↔ ‘to divide into cubes’ | /würfeln/ ∖ [_VoiceP_ Voice_ag_ [_vP_ *v* [_ResP_ __ [_Res’_ Res DP ]]
    ∼18th century

35 “lift, v.” OED Online. June 2022. Oxford University Press. https://www.oed.com/view/Entry/108138 (accessed July 26, 2022).

36 Potential examples of overt realizations of an underspecified Res(ult) head come from German prefixes like *er-*, *zer-* or *be-* (cf. Mateu 2017; but see Wilson 2021 for an alternative prepositional analysis), sometimes with idiosyncratic semantic effects as the implication of *death* in (ib).
